# Deep learning image reconstruction generates thinner slice iodine maps with improved image quality to increase diagnostic acceptance and lesion conspicuity: a prospective study on abdominal dual-energy CT

**DOI:** 10.1186/s12880-024-01334-0

**Published:** 2024-06-26

**Authors:** Jingyu Zhong, Lingyun Wang, Chao Yan, Yue Xing, Yangfan Hu, Defang Ding, Xiang Ge, Jianying Li, Wei Lu, Xiaomeng Shi, Fei Yuan, Weiwu Yao, Huan Zhang

**Affiliations:** 1 Department of Imaging, Tongren Hospital, Shanghai Jiao Tong University School of Medicine, Shanghai, 200336 China; 2 Department of Radiology, Ruijin Hospital, Shanghai Jiao Tong University School of Medicine, Shanghai, 200025 China; 3 Department of Surgery, Ruijin Hospital, Shanghai Jiao Tong University School of Medicine, Shanghai, 200025 China; 4 Computed Tomography Research Center, GE Healthcare, Beijing, 100176 China; 5 Computed Tomography Research Center, GE Healthcare, Shanghai, 201203 China; 6 Department of Materials, Imperial College London, South Kensington Campus, London, SW7 2AZ UK; 7 Department of Pathology, Ruijin Hospital, Shanghai Jiao Tong University School of Medicine, Shanghai, 200025 China

**Keywords:** Deep learning, Image reconstruction, Multidetector computed tomography, Image enhancement, Abdomen

## Abstract

**Background:**

To assess the improvement of image quality and diagnostic acceptance of thinner slice iodine maps enabled by deep learning image reconstruction (DLIR) in abdominal dual-energy CT (DECT).

**Methods:**

This study prospectively included 104 participants with 136 lesions. Four series of iodine maps were generated based on portal-venous scans of contrast-enhanced abdominal DECT: 5-mm and 1.25-mm using adaptive statistical iterative reconstruction-V (Asir-V) with 50% blending (AV-50), and 1.25-mm using DLIR with medium (DLIR-M), and high strength (DLIR-H). The iodine concentrations (IC) and their standard deviations of nine anatomical sites were measured, and the corresponding coefficient of variations (CV) were calculated. Noise-power-spectrum (NPS) and edge-rise-slope (ERS) were measured. Five radiologists rated image quality in terms of image noise, contrast, sharpness, texture, and small structure visibility, and evaluated overall diagnostic acceptability of images and lesion conspicuity.

**Results:**

The four reconstructions maintained the IC values unchanged in nine anatomical sites (all *p* > 0.999). Compared to 1.25-mm AV-50, 1.25-mm DLIR-M and DLIR-H significantly reduced CV values (all *p* < 0.001) and presented lower noise and noise peak (both *p* < 0.001). Compared to 5-mm AV-50, 1.25-mm images had higher ERS (all *p* < 0.001). The difference of the peak and average spatial frequency among the four reconstructions was relatively small but statistically significant (both *p* < 0.001). The 1.25-mm DLIR-M images were rated higher than the 5-mm and 1.25-mm AV-50 images for diagnostic acceptability and lesion conspicuity (all *P* < 0.001).

**Conclusions:**

DLIR may facilitate the thinner slice thickness iodine maps in abdominal DECT for improvement of image quality, diagnostic acceptability, and lesion conspicuity.

**Supplementary Information:**

The online version contains supplementary material available at 10.1186/s12880-024-01334-0.

## Background

Dual-energy CT (DECT) allows further material decomposition analysis to generate iodine maps for quantifying the presence of iodine-containing contrast material [1]. The iodine mapping is desirable to be used as a imaging biomarker for the detection of vascular emboli, characterization of lesions, and evaluation of treatment response [2–4]. The current application of iodine maps is mainly based on established iodine concentration thresholds; therefore, the accuracy and consistency of iodine concentration measurements has attracted attention of many researchers [5–8]. However, the application and investigation of iodine maps for diagnosis is hindered by low spatial resolution and severe image noise. The slice thickness of 5-mm and 1.25-mm is used for diagnosis in current clinical routine [9], with the 5-mm being more common for the abdomen due to the patient radiation concern. The 5-mm images display with lower image noise; however, may suffer from the low spatial resolution due to partial volume effect. In contrast, the 1.25-mm images enable higher spatial resolution for detection of more lesion details but the signal-to-noise ratio would be reduced.

It is thus very important to reconstruct iodine maps in DECT into thinner slices but with reduced image noise for iodine maps to gain clinical acceptance as interpretative images. Deep learning image reconstruction (DLIR), an algorithm uses deep convolutional neural networks, has been developed and tested for improving image quality in virtual monochromatic images (VMIs) with reduced radiation and iodine dose [10–18], and has also shown higher accuracy in iodine concentration measurements [5–8]. Nevertheless, it has not been fully investigated whether the DLIR can improve image quality of iodine maps with thinner slice thickness to increase diagnostic confidence [19].

Therefore, this prospective study is aimed to assess the hypothesis that the use of thin slice thickness (1.25-mm) iodine maps combined with DLIR in abdominal DECT can provide lower image noise, higher spatial resolution, to diagnostic acceptance and lesion conspicuity, in comparison with iodine maps reconstructed using the state-of-the-art reconstruction algorithm of adaptive statistical iterative reconstruction-V (Asir-V).

## Methods

**Fig. 1:**
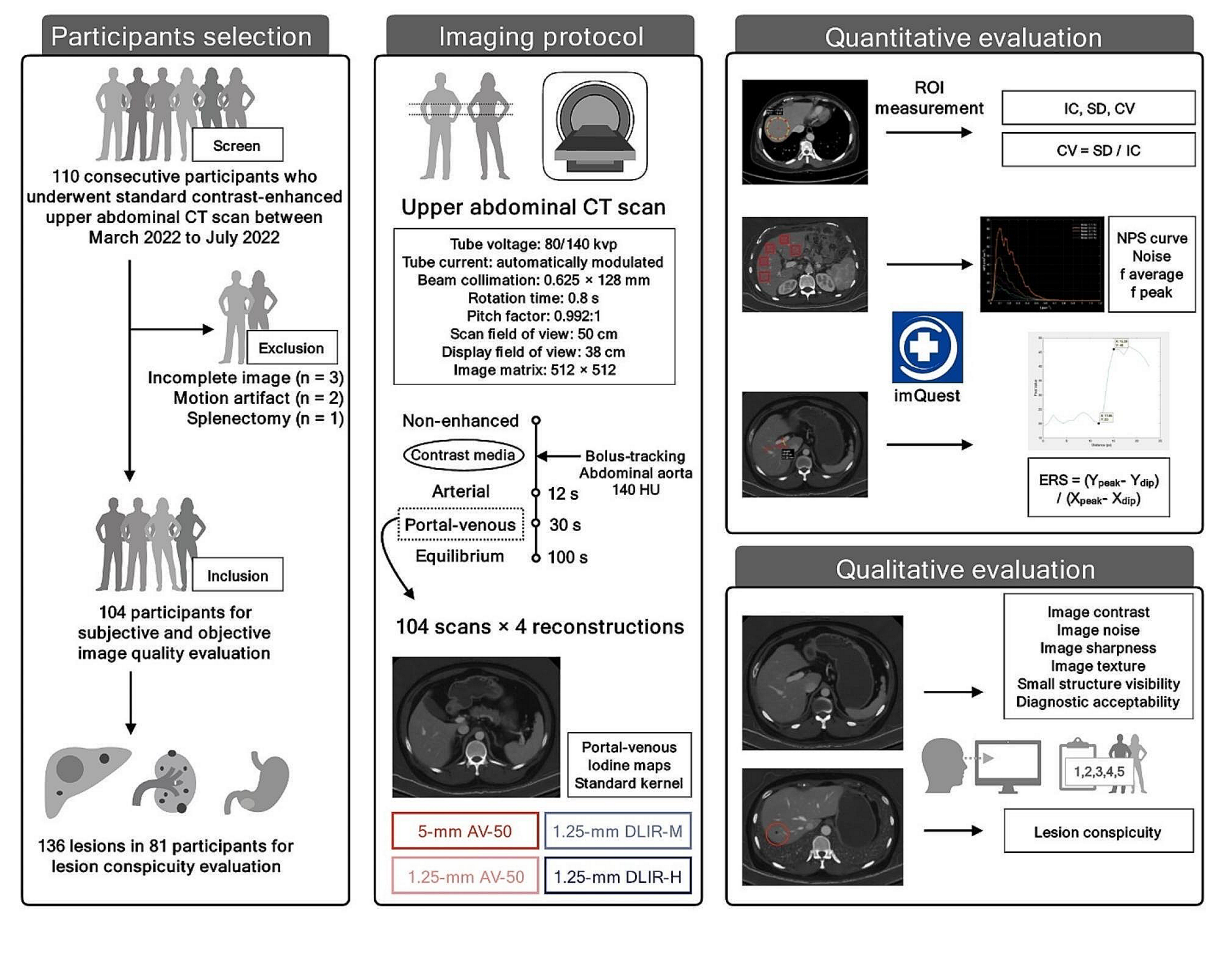
Study workflow

The local institutional ethic review board approved this study, and the written informed consents from all participants have been received. The Fig. 1 presents the workflow of current study.

### Participants

This study prospectively screened 110 consecutive participants who underwent standard abdominal contrast-enhanced CT scan for clinical purpose at our institution between March and July 2022 using a DECT scanner (Revolution Apex CT, GE Healthcare). The inclusion criteria were: (a) ≥ 18 years old; (b) scheduled to undergo abdominal contrast-enhanced CT scan for clinical purpose; (c) agree to participate the study. The exclusion criteria were: (a) severe artifacts; (b) incomplete images series or reconstruction failure; (c) lack of anatomical structure for evaluation. There were six participants excluded for: incomplete images series (*n* = 3), severe motion artifacts (*n* = 2), and splenectomy (*n* = 1), respectively. Accordingly, 104 participants were included in the study. One radiologist with 4-year-experience in radiology identified 136 lesions for analysis. The lesions were selected if they were detectable on the portal-venous scans. The largest lesion was selected when there were multiple lesions detectable in the same organ. These lesions were confirmed by an abdominal radiologist with 20-year-experience in abdominal radiology.

### Image acquisition and reconstruction

The abdominal contrast-enhanced DECT scans were conducted using a typical abdominal protocol. The DECT scans were performed using a fast-kilovoltage-switching technique between 80 and 140 kVp, with an automatic tube current (GSI Assist, GE Healthcare), 0.625 × 128 mm of beam collimation, 0.8 s of rotation time, 0.992:1 of pitch factor, 50 cm of scan field of view, 38 cm of display field of view, and 512 × 512 of image matrix. A nonionic contrast media was administered intravenously (approximately 520 mgI/kg body weight) within a fixed duration of 30 s. The real-time fluoroscopic monitoring (140 kVp, 20 mA) was initiated 10 s after the contrast injection. After the bolus-tracking program (SmartPrep, GE Healthcare) detected an enhancement over the threshold of 140 HU in the abdominal aorta, the arterial, portal-venous, and equilibrium scans were initiated with additional delays of 12, 30, and 100 s. The delay duration in this protocol was optimized by the local institution.

The raw data of portal-venous scans were used for reconstruction. There were four series of iodine maps for each participant reconstructed using a vendor-specific workstation (advantage workstation, AW version 4.7, GE Healthcare): 5-mm (thick slice) and 1.25-mm (thin slice) using Asir-V with 50% blending (AV-50), and 1.25-mm using DLIR with medium (DLIR-M), and high strength (DLIR-H), all applying the standard kernel. The slice thickness and blending ratio of Asir-V is determined according to the institutional clinical routine. The 5-mm and 1.25-mm AV-50 images were used as reference for image spatial resolution and noise, respectively. Our pilot study suggested that only the 1.25-mm DLIR-M and DLIR-H images have potential in improving both image noise and spatial resolution (Supplementary Note S1). However, the DLIR-L did not show advantage compared to the routine 1.25-mm or 5-mm AV-50 images. Therefore, the 1.25-mm DLIR-L images were not reconstructed.

### Quantitative image evaluation

A radiologist with 4-year-experience in radiology conducted the quantitative image evaluation using the open-source imQuest software version 7.1 (Duke University; https://deckard.duhs.duke.edu/~samei/tg233.html) (Supplementary Note S2) [5, 8, 9, 13]. Regions of interest (ROI) were selected on the 5 mm AV-50 images. The corresponding images in thinner slices in other reconstructions were linked, to ensure the identical ROIs were used for evaluation. The iodine concentration (IC) and its standard deviation (SD) of nine anatomic structures were measured. The coefficient of variation (CV) for each structure was calculated to evaluate the extent of variability by dividing the mean IC by the SD over the patient cohort. The image noise magnitude was evaluated using noise power spectrum (NPS) by placing ROIs on the relatively homogenous regions of the liver. The NPS curve, NPS noise, NPS noise peak, the average spatial frequency and the peak spatial frequency were generated. The image sharpness was evaluated using edge rise slope (ERS). The ERS was calculated by dividing the IC difference between the last dip and the first peak on the rising IC curve by the distance between these two points. The ERS was measured using a selected axial plane that presents a running portal vein. Ten participants were randomly selected for repeated measurements by the same radiologist and by another radiologist with 5-year-experience in radiology two weeks after the first readout. These repeated measurements were used to calculate the intra- in inter-rater quantitative measurement variabilities, respectively.

### Qualitative image evaluation

Five radiologists with 1- to 6-year-experience in radiology performed the qualitative image quality assessment (Supplementary Note S3) [6, 7, 9, 11–16, 18]. The readers independently rated image quality in terms of image noise, contrast, sharpness, texture, small structure visibility, and evaluated overall diagnostic acceptability of images and lesion conspicuity. A value of more than 3 was defined as an image quality satisfactory for clinical use. The readers should detect the lesions for evaluation by themselves but were instructed what lesions to rate. All the images were randomly presented to the readers without reconstruction parameters. The images were shown with a window width of 15.0 mgI/mL and window level of 5.0 mgI/mL, using the same settings for daily image interpretation at the reading room. There were no time limits for the evaluation, and the readers can view the images with the window width and level as well as distance they like. Two weeks after the first readout, a radiologist with 5-year- experience in radiology repeated all the assessment of images and lesions. The intra- in inter-rater qualitative assessment variabilities were calculated using assessments of five raters and the repeated assessments by this rater, respectively.

### Statistical analysis

We applied R language version 4.1.3 (https://www.r-project.org/) with related packages within RStudio version 1.4.1106 (https://www.rstudio.com/) (Supplementary Note S4) [20] for statistical analysis. The difference of among the reconstruction algorithms were performed by using repeated-measure analysis of variance (ANOVA) for quantitative metrics and Friedman test for qualitative metrics, respectively. Once there was an overall significant difference, the *post hoc* pairwise comparisons between groups would be conducted with Bonferroni correction. For the lesion conspicuity, subgroup analysis was performed according to (1) location of lesion, (2) the largest diameter of lesions, and (3) the presentation of lesions compared to surrounding tissue. The statistical analysis was two-tailed and the alpha value was set at 0.05. The agreements of quantitative evaluations were evaluated by using an intraclass correlation coefficient (ICC) of single measurement, absolute agreement, and two-way random-effects model [21]. For qualitative evaluations, the intra-rater and inter-rater agreements were evaluated by using the weighted kappa statistic for and the Kendall’s W statistics, respectively [22]. According to our pilot study, the *a priori* sample size estimation yielded a size of 22 participants for a power of 0.85, when alpha was 0.05. With 104 participants, the *post hoc* power calculation resulted in 1-beta values > 0.995, when alpha was 0.05, indicating an efficient statistical power [23].

## Results

### Characteristics of participants and lesions

Our study included 104 participants (62 men; mean age ± standard deviation, 56.8 ± 13.1 years; median 58, range, 24 to 80 years) with 136 lesions (mean size, 14.5 ± 14.1 mm; median 11, range, 3 to 107 mm) for analysis (Table 1 and Supplementary Table S1).

**Table 1:** Participant and lesion characteristics

Characteristics	Data
Number of participants	104
Age, year, mean ± SD, median (range)	56.8 ± 13.1, 58 (24 to 80)
Gender, n (%)	
Male	62 (60)
Female	42 (40)
Clinical purpose, n (%)	
Cancer stating	71 (68)
Cancer Surveillance	33 (32)
Number of lesions	136
No. of lesions of each participant, mean ± SD, median (range)	1.3 ± 0.9, 1 (0 to 3)
Largest diameter of lesions, millimeters, mean ± SD, median (range)	14.5 ± 14.1, 11 (3 to 107)
Presentation of lesions compared to surrounding tissue, n (%)	
Lower	109 (80)
Mixed	18 (13)
Higher	9 (7)
Location of lesions, n (%)	
Right lobe of Liver	48 (35)
Right kidney	32 (24)
Left kidney	24 (18)
Left lobe of Liver	19 (14)
Gallbladder	4 (3)
Spleen	4 (3)
Stomach	3 (2)
Pancreas	2 (1)

*Note* SD = standard deviation

### Quantitative evaluation results

**Fig. 2:**
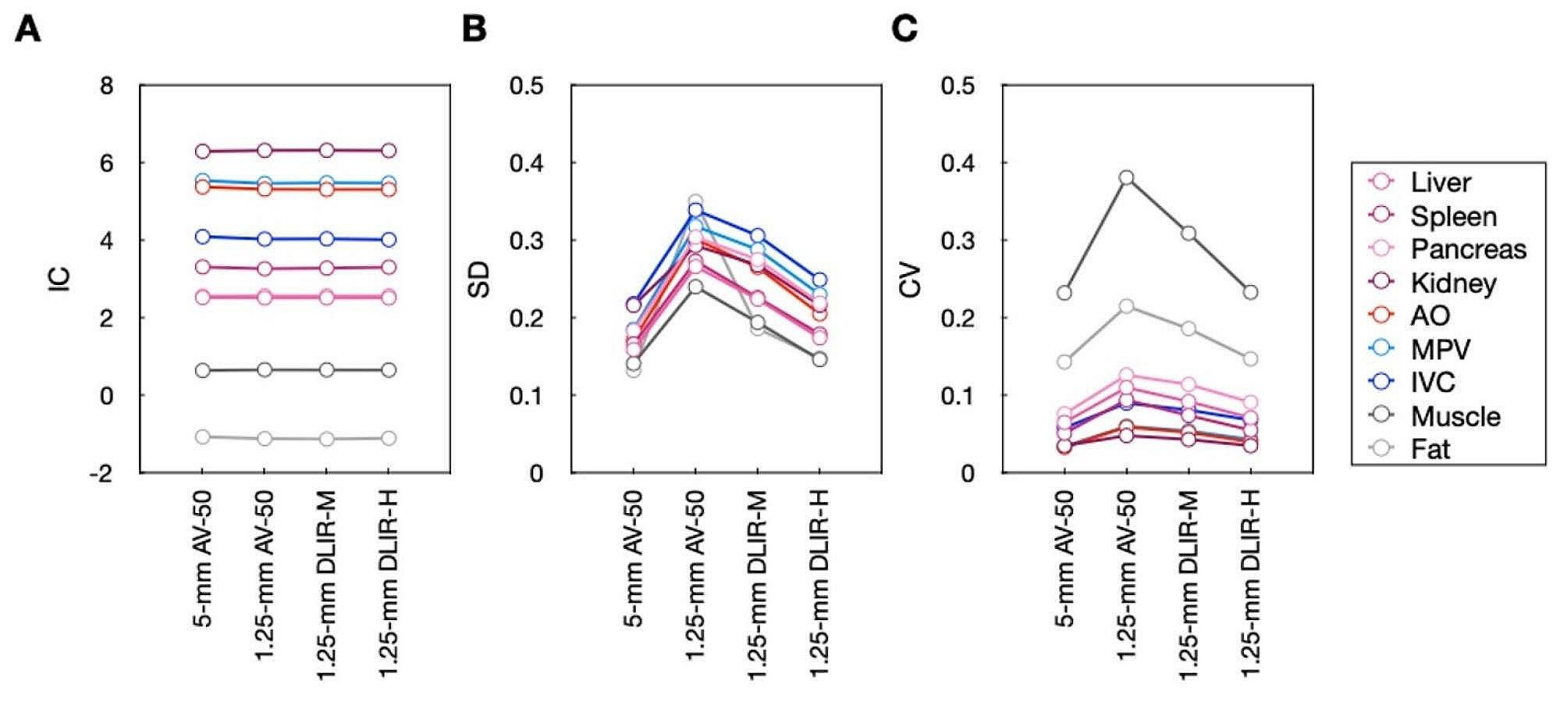
Iodine concentration and variability. (**A**) IC values, (**B**) SD values, and (**C**) CV values of nine anatomical sites obtained using the four different image reconstruction algorithms

The intra-rater and inter-rater agreements were excellent for IC of liver (ICC 0.938–0.939) and SD of liver IC (ICC 0.921–0.967) measurements (Supplementary Table S2). The effect sizes of these quantitative evaluation metrics between the different reconstruction algorithms are calculated (Supplementary Table S3). The IC values remained stable among the four series with different reconstruction algorithms (all *p* > 0.05) (Table 2; Fig. 2). The CV values of nine anatomical sites on the thin slice DLIR-M and DLIR-H images were lower than that on the thin slice AV-50 (all *p* < 0.001), with that of the thin DLIR-H images being the lowest among the three groups. The CV values of kidney, psoas major, and abdominal subcutaneous fat on the thin slice DLIR-H were similar to that on the thick slice AV-50 images (all *p* > 0.999), but those of other anatomical sites were slightly higher (all other *p* < 0.001).

**Table 2:** Iodine concentration and variability

	5-mm AV-50	1.25-mm AV-50	1.25-mm DLIR-M	1.25-mm DLIR-H	*p*	p1	p2	p3	p4	p5	p6
Iodine concentration and variability											
Liver											
IC, mgI/mL	2.520 ± 0.524	2.509 ± 0.520	2.510 ± 0.521	2.509 ± 0.519	0.757	n. a.	n. a.	n. a.	n. a.	n. a.	n. a.
SD, mgI/mL	0.159 ± 0.037	0.266 ± 0.048	0.223 ± 0.043	0.174 ± 0.041	< 0.001	< 0.001	< 0.001	< 0.001	< 0.001	< 0.001	< 0.001
CV	0.065 ± 0.017	0.109 ± 0.025	0.091 ± 0.021	0.071 ± 0.018	< 0.001	< 0.001	< 0.001	< 0.001	< 0.001	< 0.001	< 0.001
Spleen											
IC, mgI/mL	3.311 ± 0.645	3.262 ± 0.744	3.28 ± 0.707	3.305 ± 0.647	0.502	n. a.	n. a.	n. a.	n. a.	n. a.	n. a.
SD, mgI/mL	0.166 ± 0.047	0.272 ± 0.049	0.226 ± 0.042	0.178 ± 0.041	< 0.001	< 0.001	< 0.001	< 0.001	< 0.001	< 0.001	< 0.001
CV	0.051 ± 0.014	0.094 ± 0.084	0.073 ± 0.037	0.055 ± 0.012	< 0.001	< 0.001	< 0.001	< 0.001	< 0.001	< 0.001	< 0.001
Pancreas											
IC, mgI/mL	2.557 ± 0.654	2.569 ± 0.647	2.567 ± 0.651	2.568 ± 0.65	0.324	n. a.	n. a.	n. a.	n. a.	n. a.	n. a.
SD, mgI/mL	0.183 ± 0.053	0.303 ± 0.067	0.275 ± 0.063	0.218 ± 0.058	< 0.001	< 0.001	< 0.001	< 0.001	< 0.001	< 0.001	< 0.001
CV	0.075 ± 0.029	0.126 ± 0.045	0.114 ± 0.042	0.091 ± 0.038	< 0.001	< 0.001	< 0.001	< 0.001	< 0.001	< 0.001	< 0.001
Kidney											
IC, mgI/mL	6.294 ± 1.122	6.316 ± 1.102	6.319 ± 1.102	6.312 ± 1.105	0.265	n. a.	n. a.	n. a.	n. a.	n. a.	n. a.
SD, mgI/mL	0.215 ± 0.099	0.293 ± 0.094	0.267 ± 0.088	0.215 ± 0.079	< 0.001	< 0.001	< 0.001	**> 0.999**	< 0.001	< 0.001	< 0.001
CV	0.035 ± 0.016	0.047 ± 0.016	0.043 ± 0.015	0.034 ± 0.012	< 0.001	< 0.001	< 0.001	**> 0.999**	< 0.001	< 0.001	< 0.001
Abdominal aorta											
IC, mgI/mL	5.374 ± 0.998	5.314 ± 0.992	5.308 ± 0.995	5.307 ± 0.996	0.532	n. a.	n. a.	n. a.	n. a.	n. a.	n. a.
SD, mgI/mL	0.170 ± 0.044	0.301 ± 0.050	0.265 ± 0.046	0.205 ± 0.040	< 0.001	< 0.001	< 0.001	< 0.001	< 0.001	< 0.001	< 0.001
CV	0.032 ± 0.011	0.058 ± 0.015	0.051 ± 0.013	0.039 ± 0.010	< 0.001	< 0.001	< 0.001	< 0.001	< 0.001	< 0.001	< 0.001
Main portal vein											
IC, mgI/mL	5.504 ± 1.025	5.463 ± 1.035	5.479 ± 1.037	5.472 ± 1.040	0.383	n. a.	n. a.	n. a.	n. a.	n. a.	n. a.
SD, mgI/mL	0.185 ± 0.070	0.317 ± 0.066	0.287 ± 0.064	0.230 ± 0.059	< 0.001	< 0.001	< 0.001	< 0.001	< 0.001	< 0.001	< 0.001
CV	0.034 ± 0.014	0.059 ± 0.015	0.053 ± 0.014	0.043 ± 0.012	< 0.001	< 0.001	< 0.001	< 0.001	< 0.001	< 0.001	< 0.001
Inferior vena cava											
IC, mgI/mL	4.091 ± 0.961	4.03 ± 0.938	4.035 ± 0.938	4.014 ± 0.987	0.327	n. a.	n. a.	n. a.	n. a.	n. a.	n. a.
SD, mgI/mL	0.217 ± 0.083	0.338 ± 0.075	0.305 ± 0.072	0.248 ± 0.076	< 0.001	< 0.001	< 0.001	< 0.001	< 0.001	< 0.001	< 0.001
CV	0.057 ± 0.031	0.089 ± 0.033	0.081 ± 0.031	0.068 ± 0.035	< 0.001	< 0.001	< 0.001	< 0.001	< 0.001	< 0.001	< 0.001
Psoas major											
IC, mgI/mL	0.638 ± 0.132	0.660 ± 0.134	0.656 ± 0.133	0.652 ± 0.133	0.416	n. a.	n. a.	n. a.	n. a.	n. a.	n. a.
SD, mgI/mL	0.141 ± 0.024	0.239 ± 0.035	0.194 ± 0.028	0.146 ± 0.024	< 0.001	< 0.001	< 0.001	0.023	< 0.001	< 0.001	< 0.001
CV	0.231 ± 0.068	0.380 ± 0.108	0.308 ± 0.081	0.233 ± 0.063	< 0.001	< 0.001	< 0.001	**> 0.999**	< 0.001	< 0.001	< 0.001
Abdominal subcutaneous fat											
IC, mgI/mL	-1.071 ± 0.265	-1.119 ± 0.256	-1.131 ± 0.250	-1.107 ± 0.340	0.994	n. a.	n. a.	n. a.	n. a.	n. a.	n. a.
SD, mgI/mL	0.131 ± 0.045	0.215 ± 0.066	0.185 ± 0.071	0.147 ± 0.064	< 0.001	< 0.001	< 0.001	< 0.001	< 0.001	< 0.001	< 0.001
CV	-0.143 ± 0.105	-0.215 ± 0.128	-0.186 ± 0.134	-0.147 ± 0.121	< 0.001	< 0.001	< 0.001	**> 0.999**	< 0.001	< 0.001	< 0.001

*Note* Data were presented as mean ± standard deviation. p = p value for repeated-measure analysis of variance. p1 to p6 were p values for post hoc pairwise comparisons using Bonferroni correction, the p values presented were presented as adjusted p values

**Fig. 3:**
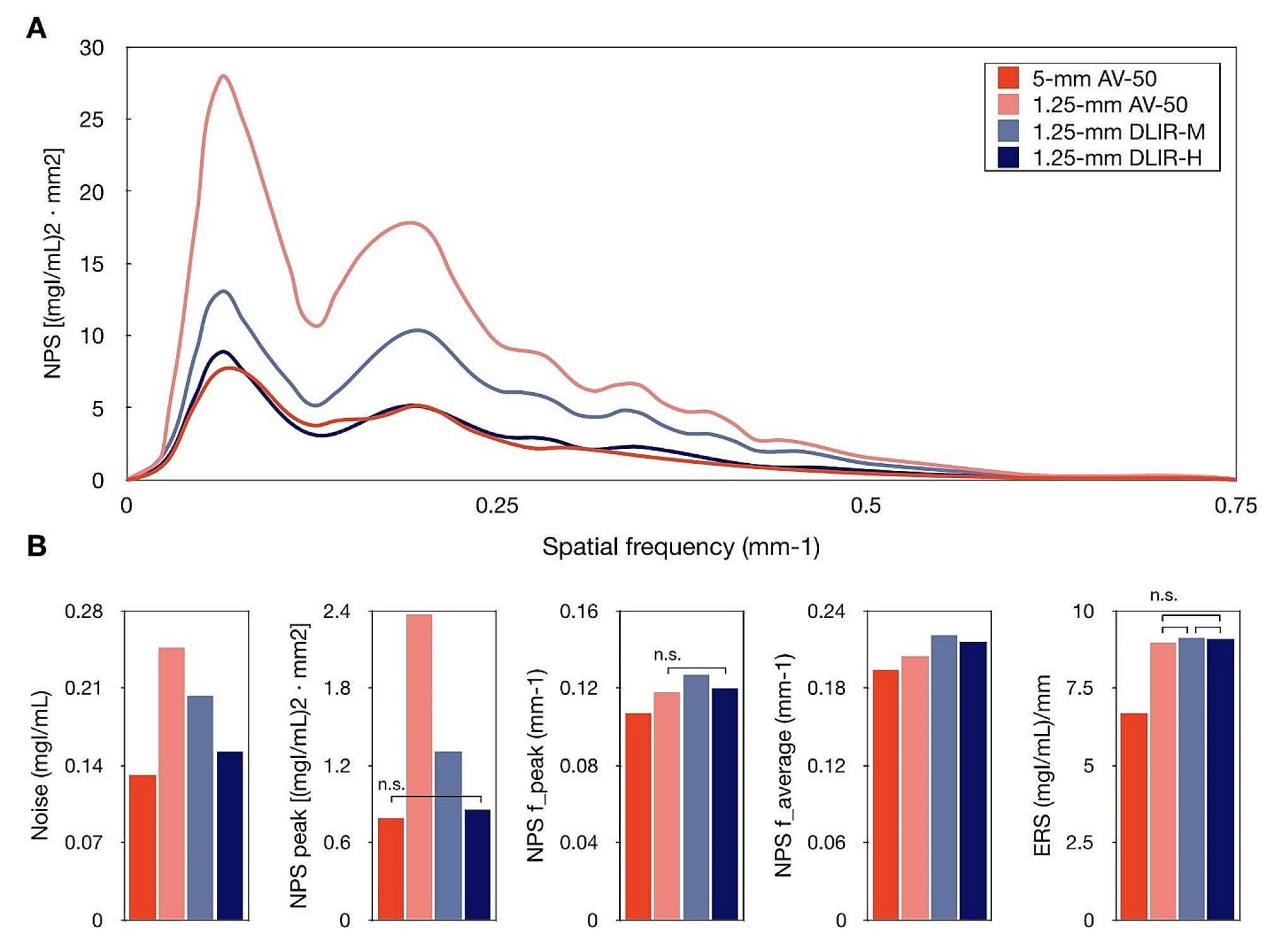
NPS and ERS assessment. (**A**) NPS curves obtained with the four different image reconstruction algorithms using homogenous region of the liver of a patient. (**B**) The NPS and ERS metrics among the four different image reconstruction algorithms. n. s. indicates non-significant post hoc comparison results using Bonferroni method

The intra-rater and inter-rater agreements were moderate to excellent for NPS metrics (ICC 0.731–0.960) and ERS (ICC 0.933–0.964) measurements (Supplementary Table S2). The noise on the thin slice DLIR-H images were lower than thin slice AV-50 (*p* < 0.001), and thin slice DLIR-M images (*p* < 0.001), but were higher than that on the thick slice AV-50 images (*p* < 0.001) (Table 3; Fig. 3). The noise peaks of DLIR-H images were similar to that of the thick slice AV-50 images (*p* = 0.150). The peak spatial frequency and average spatial frequency varied among the four reconstruction algorithms, except for the peak spatial frequency between thin slice AV-50, and thin slice DLIR-H images (*p* = 0.210). However, the difference of the peak and average spatial frequency among the four reconstruction algorithms were relatively small. The ERS values on the thin slice AV-50, thin slice DLIR-M, and thin slice DLIR-H images were higher than that on thick slice AV-50 images (all *P* < 0.001), but the difference in ERS values among the thin slice images could not be identified (all *p* > 0.999).

**Table 3:** NPS and ERS assessment

	5-mm AV-50	1.25-mm AV-50	1.25-mm DLIR-M	1.25-mm DLIR-H	*p*	p1	p2	p3	p4	p5	p6
Noise, mgI/mL	0.130 ± 0.017	0.246 ± 0.026	0.202 ± 0.022	0.152 ± 0.019	< 0.001	< 0.001	< 0.001	< 0.001	< 0.001	< 0.001	< 0.001
Noise peak, (mgI/mL)^2^ · mm^2^	0.788 ± 0.331	2.371 ± 0.883	1.308 ± 0.542	0.859 ± 0.445	< 0.001	< 0.001	< 0.001	**0.150**	< 0.001	< 0.001	< 0.001
f_peak_, mm^− 1^	0.106 ± 0.028	0.117 ± 0.031	0.126 ± 0.040	0.120 ± 0.041	< 0.001	0.001	< 0.001	0.003	< 0.001	**0.210**	< 0.001
f_average_, mm^− 1^	0.193 ± 0.029	0.205 ± 0.022	0.220 ± 0.021	0.215 ± 0.025	< 0.001	< 0.001	< 0.001	< 0.001	< 0.001	< 0.001	< 0.001
ERS, (mgI/mL)/mm	6.703 ± 2.880	8.997 ± 4.474	9.132 ± 4.554	9.103 ± 4.545	< 0.001	< 0.001	< 0.001	< 0.001	**> 0.999**	**> 0.999**	**> 0.999**

*Note* Data were presented as mean ± standard deviation. p = p value for repeated-measure analysis of variance. p1 to p6 were p values for post hoc pairwise comparisons using Bonferroni correction, the p values presented were presented as adjusted p values

### Qualitative evaluation results

**Fig. 4:**
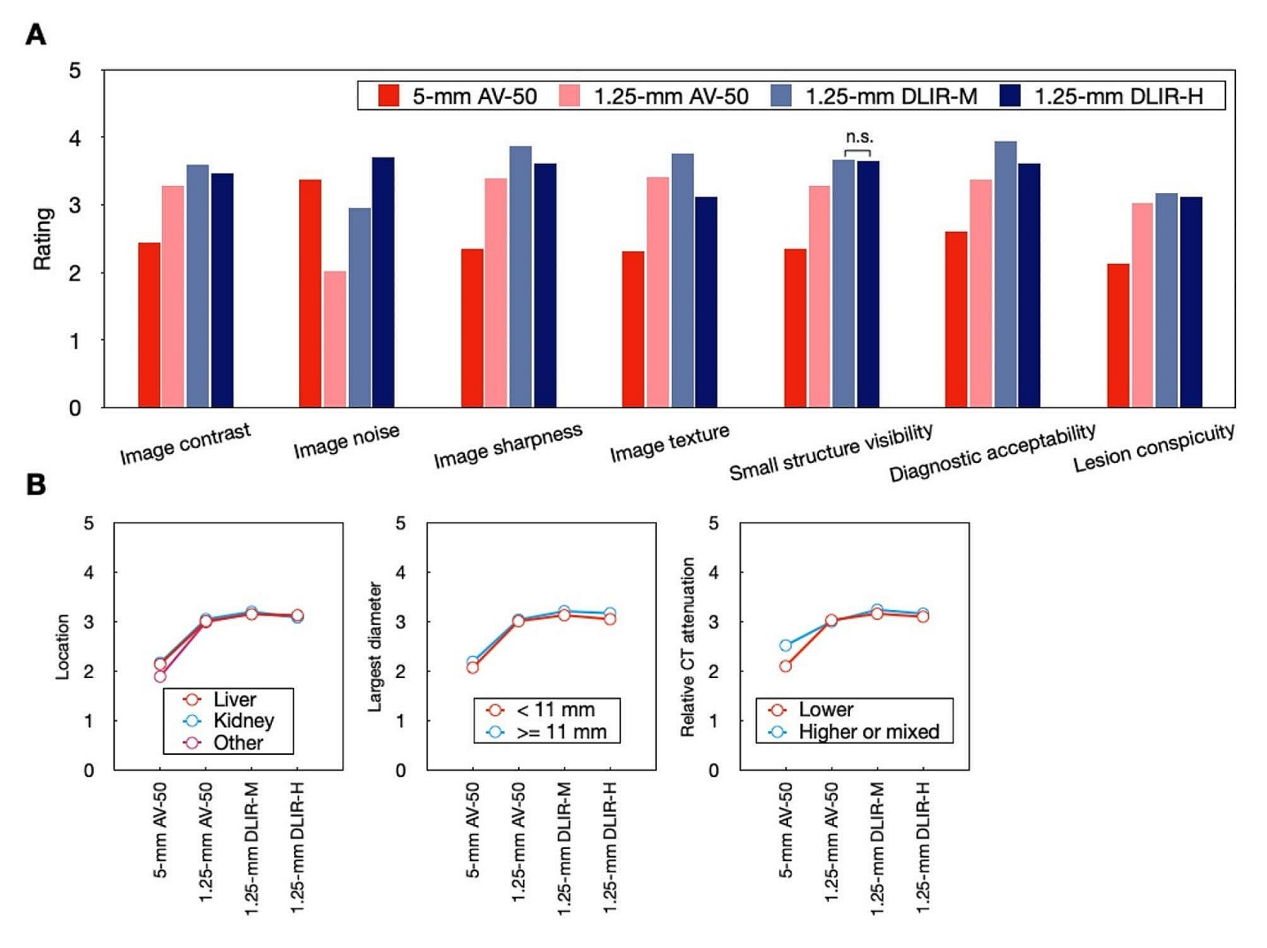
Qualitative image quality and lesion conspicuity rating. Bar plots for image noise, image contrast, image sharpness, small structure visibility, diagnostic acceptability of participants (*n* = 104), and lesion conspicuity of evaluated lesions (*n* = 136) obtained with four image reconstruction algorithms. n. s. indicates non-significant post hoc comparison results using Bonferroni method

The intra-rater and inter-rater agreements were moderate to good (weighted kappa statistic 0.546–0.769; Kendall’s W statistic 0.536–0.717) for qualitative analysis (Supplementary Table S2). The effect sizes of these qualitative evaluation metrics between the different reconstruction algorithms are calculated (Supplementary Table S4). The thin slice DLIR-H images were rated the highest for image noise (*p* < 0.001) and presented comparable ratings for small structure visibility as the thin slice DLIR-M images (*p* > 0.999) (Table 4; Fig. 4). The thin slice DLIR-M images were rated the highest in terms of image contrast, sharpness, and texture, and gained the highest diagnostic acceptance among four image reconstruction algorithms (all *p* < 0.001). The thin slice AV-50 images suffered from undiagnostic image noise, while the thick slice AV-50 only showed advantages in image noise. Both of the AV-50 images were less acceptable for diagnosis than DLIR images (all *p* < 0.001).

**Table 4:** Qualitative image quality rating

	5-mm AV-50	1.25-mm AV-50	1.25-mm DLIR-M	1.25-mm DLIR-H	*p*	p1	p2	p3	p4	p5	p6
Image contrast	2.43 ± 0.25	3.29 ± 0.24	3.59 ± 0.26	3.46 ± 0.28	< 0.001	< 0.001	< 0.001	< 0.001	< 0.001	0.002	0.002
Image noise	3.37 ± 0.21	2.01 ± 0.17	2.95 ± 0.22	3.70 ± 0.26	< 0.001	< 0.001	< 0.001	< 0.001	< 0.001	< 0.001	< 0.001
Image sharpness	2.35 ± 0.22	3.39 ± 0.22	3.87 ± 0.24	3.61 ± 0.31	< 0.001	< 0.001	< 0.001	< 0.001	< 0.001	< 0.001	< 0.001
Image texture	2.31 ± 0.23	3.40 ± 0.24	3.75 ± 0.25	3.11 ± 0.32	< 0.001	< 0.001	< 0.001	< 0.001	< 0.001	< 0.001	< 0.001
Small structure visibility	2.35 ± 0.25	3.27 ± 0.26	3.65 ± 0.31	3.64 ± 0.28	< 0.001	< 0.001	< 0.001	< 0.001	< 0.001	< 0.001	**> 0.999**
Diagnostic acceptability	2.61 ± 0.25	3.38 ± 0.24	3.93 ± 0.18	3.61 ± 0.27	< 0.001	< 0.001	< 0.001	< 0.001	< 0.001	0.004	< 0.001

*Note* Data were presented as mean ± standard deviation. p = p value for Friedman test. p1 to p6 were p values for post hoc pairwise comparisons using Bonferroni correction, the p values presented were presented as adjusted p values

**Fig. 5:**
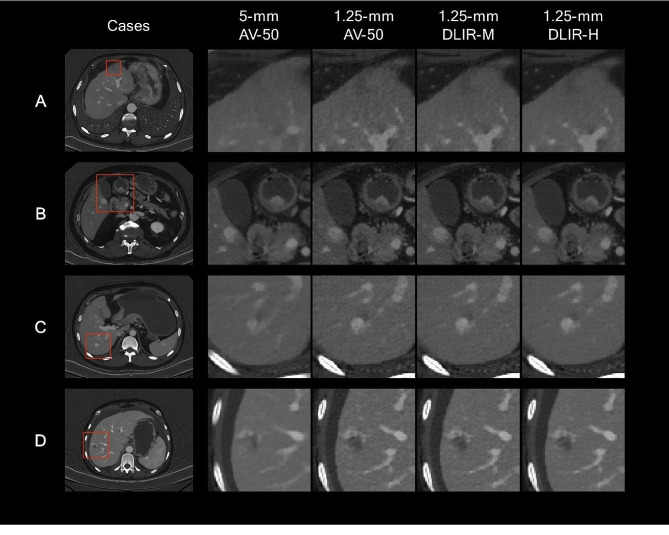
Four examples of abdominal CT studies. These portal-venous phase abdominal CT studies were reconstructed into iodine maps using 5-mm AV-50, 1.25-mm AV-50, 1.25-mm DLIR-M, and 1.25-mm DLIR-H algorithms; and present with the same windowing (width of 15.0 mgI/mL, level of 5.0 mgI/mL). (A) A patient underwent CT scan for cancer staging. A low-density hepatic lesion was better visualized in 1.25-mm images than the 5-mm AV-50 image. The 1.25-mm DLIR image better balanced the image noise and spatial resolution. (**B**) A patient underwent CT scan for cancer staging. The enhanced hepatic lesion can be detected in all the images, while the details of the texture and boundary were better visualized in 1.25-mm images. The lesion of the stomach can be detected in all the images, but the enhanced range was better displayed with lower image noise in 1.25-mm DLIR-H images, potentially allowed more accurate staging. (**C**) A patient underwent CT scan for hepatic lesion. Compared with the 5-mm AV-50 image, the 1.25-mm images better showed the enhance pattern of the lesion. The 1.25-mm DLIR-H images presented the lesion with more details and lower image noise. (**D**) A patient underwent CT scan for hepatic lesion. A hepatic lesion with surrounding enhancement was detected. The lesion texture of is better depicted with 1.25-mm images. The 1.25-mm DLIR-H images best balanced the image noise, and spatial resolution

**Fig. 6:**
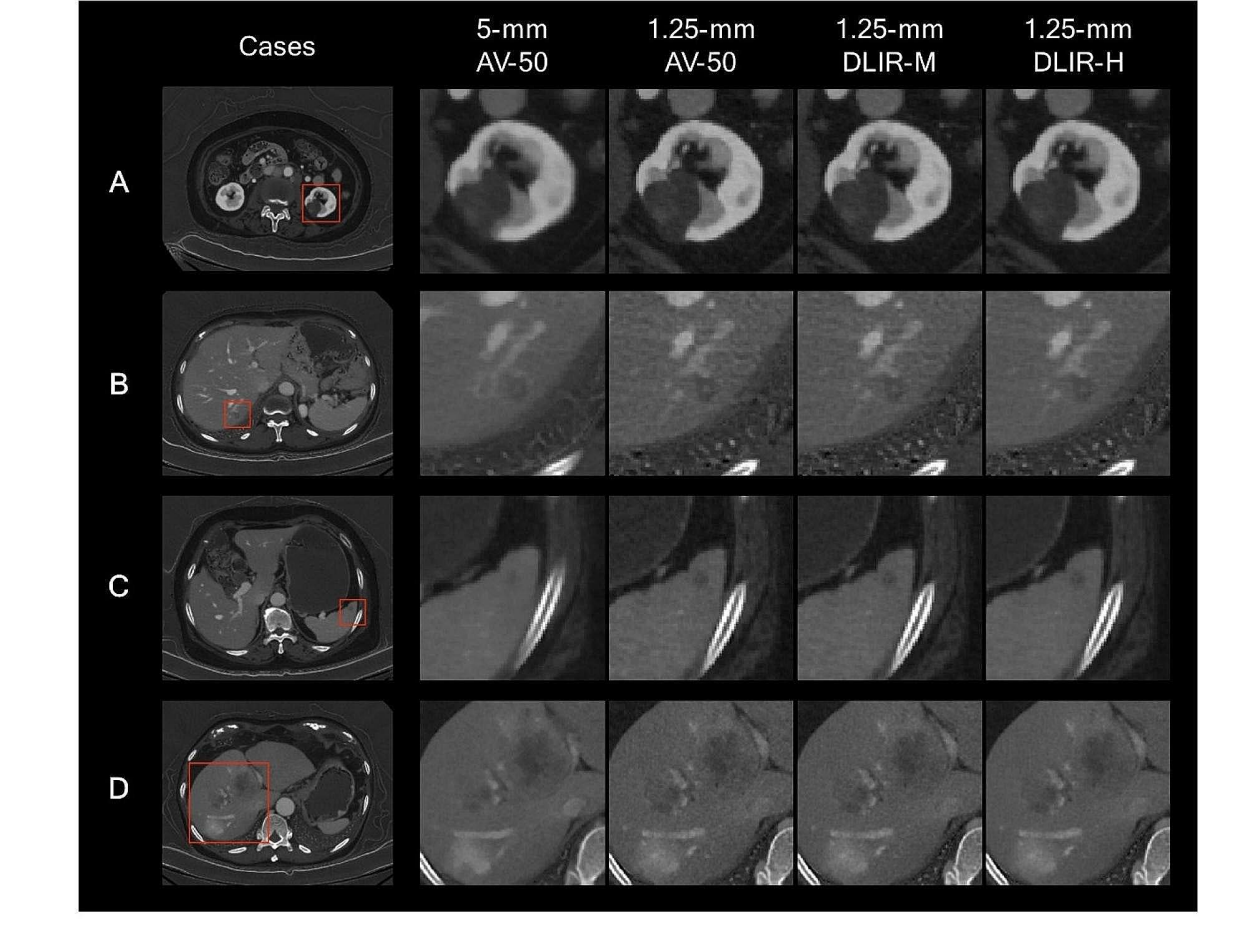
Four examples of abdominal CT studies with measurements. These portal-venous phase abdominal CT studies were reconstructed into iodine maps using 5-mm AV-50, 1.25-mm AV-50, 1.25-mm DLIR-M, and 1.25-mm DLIR-H algorithms, respectively; and present with the same windowing (width of 15.0 mgI/mL, level of 5.0 mgI/mL). (**A**) A patient was scanned for a suspected left renal lesion. With 1.25-mm images, the internal enhancement was better detected. The difference of iodine concentration between the low-density compartment and subtle enhancement was more obvious with 1.25-mm AV-50 (1.14 mgI/mL) and 1.25-mm DLIR-H (1.04 mgI/mL) images, than 5-mm AV-50 (0.68 mgI/mL) image, which suffered from the partial volume effects. (**B**) A patient was scanned for cancer staging. The hepatic lesion with subtle circular and center enhancement in with 5-mm AV-50 image was suspected to be a metastasis lesion. The 1.25-mm images better visualized the boundary of lesion, and the subtle circular enhancement was excluded. The detailed center enhancement in the lesion can be better depicted in 1.25-mm images. The ERS of border of the enhancement was higher in 1.25-mm AV-50 [0.437 (mgI/mL)/mm] image than 5-mm AV-50 [0.262 (mgI/mL)/mm] image, and was even higher in 1.25 DLIR-H [0.655 (mgI/mL)/mm] image. The follow-up scans indicated that the lesion is benign. (**C**) A patient was scanned for a lesion in spleen. The difference of iodine concentration between the low-density lesion and spleen parenchyma was more obvious with 1.25-mm AV-50 (1.14 mgI/mL) and 1.25-mm DLIR-H (1.04 mgI/mL) images, than 5-mm AV-50 (0.60 mgI/mL) image. The ERS of the lesion boundary was higher in 1.25-mm AV-50 [0.334 (mgI/mL)/mm] and 1.25 DLIR-H [0.359 (mgI/mL)/mm] images than 5-mm AV-50 [0.164 (mgI/mL)/mm] image. The 1.25-mm DLIR-H image with lower noise improved the diagnostic confidence of a cyst. (**D**) A patient with gastric cancer was scanned for hepatic metastases. With the 1.25-mm images, the left boundary of the lesion was clearer than the 5-mm AV-50 images. The ERS of the subtle left boundary was higher in in 1.25-mm DLIR-H [0.586 (mgI/mL)/mm] image than 1.25 AV-50 [0.373 (mgI/mL)/mm] image, and lowest in 5-mm AV-50 [0.1.66 (mgI/mL)/mm] image. It was hard to measure the size of the lesion in 5-mm image, while it can be easier to be measured in 1.25-mm images, to guide later treatment selections

For lesion conspicuity, the thin slice images showed higher acceptance than the thick slice AV-50 images by five readers (all *p* < 0.001) (Table 5; Fig. 4). Among the thin slice images, the thin slice DLIR-M gained higher rating than the thin slice AV-50 and thin slice DLIR-H (both *p* < 0.001). The subgroup analysis for lesion conspicuity suggested that the thin slice DLIR-M images were the most acceptable for the readers, while the thin slice DLIR-H images did not show significant improvements in lesion conspicuity compared to thin slice AV-50. The representative cases for lesion characterization are shown in Figs. 5 and 6, and Supplementary Figure S1.

**Table 5:** Lesion conspicuity rating

	5-mm AV-50	1.25-mm AV-50	1.25-mm DLIR-M	1.25-mm DLIR-H	*p*	p1	p2	p3	p4	p5	p6
Overall	2.13 ± 0.47	3.03 ± 0.32	3.17 ± 0.34	3.11 ± 0.34	< 0.001	< 0.001	< 0.001	< 0.001	< 0.001	**0.145**	0.029
Location of lesion											
Liver (*n* = 67)	2.14 ± 0.48	3.01 ± 0.35	3.15 ± 0.35	3.13 ± 0.34	< 0.001	< 0.001	< 0.001	< 0.001	< 0.001	**0.210**	**> 0.999**
Kidney (*n* = 56)	2.17 ± 0.44	3.05 ± 0.30	3.20 ± 0.33	3.09 ± 0.33	< 0.001	< 0.001	< 0.001	< 0.001	< 0.001	**> 0.999**	**0.050**
Other (*n* = 13)	1.89 ± 0.50	2.99 ± 0.30	3.17 ± 0.41	3.09 ± 0.36	< 0.001	< 0.001	< 0.001	< 0.001	**0.664**	**> 0.999**	**> 0.999**
Largest diameter of lesion											
< 11 mm (*n* = 66)	2.07 ± 0.43	3.01 ± 0.32	3.13 ± 0.33	3.05 ± 0.34	< 0.001	< 0.001	< 0.001	< 0.001	< 0.001	**> 0.999**	**0.091**
>= 11 mm (*n* = 70)	2.19 ± 0.50	3.04 ± 0.33	3.21 ± 0.35	3.17 ± 0.34	< 0.001	< 0.001	< 0.001	< 0.001	< 0.001	**0.156**	**0.697**
Presentation of lesions compared to surrounding tissue											
Lower (*n* = 109)	2.10 ± 0.46	3.03 ± 0.31	3.16 ± 0.33	3.10 ± 0.34	< 0.001	< 0.001	< 0.001	< 0.001	< 0.001	**0.559**	**0.165**
Higher or mixed (*n* = 27)	2.52 ± 0.47	3.00 ± 0.38	3.24 ± 0.38	3.16 ± 0.37	< 0.001	< 0.001	< 0.001	< 0.001	< 0.001	**0.550**	**0.347**

*Note* Data were presented as mean ± standard deviation. p = p value for Friedman test. p1 to p6 were p values for post hoc pairwise comparisons using Bonferroni correction, the p values presented were presented as adjusted p values

## Discussion

The current study evaluated the improvements in image quality, diagnostic acceptance, and lesion conspicuity of using thinner slice iodine maps combined with DLIR algorithm. The thin slice DLIR images provided stable IC measurement compared to the conventional Asir-V image reconstruction algorithm and showed lower CV values than that of thin slice AV-50 to allow accurate and consistent iodine quantification. The thin slice DLIR significantly reduced the image noise compared to thin slice AV-50, while provided higher spatial resolution with thinner slice thickness compared to thick slice AV-50. The subjective evaluation showed higher diagnostic acceptance and higher lesion conspicuity with thin slice DLIR images compared to thick slice AV-50 images, indicating the potential of thin slice iodine maps with DLIR for clinical diagnostic purpose.

The previous phantom studies have demonstrated that DECT scanners using a fast-kilovoltage-switching mode with DLIR can provide possible small improvement in iodine quantification accuracy compared with the Asir-V [5, 8]. The clinical study further confirmed the potential of DLIR in reducing image noise as well as variability of IC values compared to Asir-V [6–8]. However, the studies only investigated the IC accuracy and image quality at one slice thickness of 5-mm [5] or thin slice [6–8]. As the current clinical standard of obtaining iodine maps is still iterative reconstruction with relative thick slice thickness, we further investigated the influence of slice thickness on the iodine quantification. Our results suggested that the DLIR allows thinner slice thickness with consistent IC values with lower variability, indicating the generalizability of quantitative thresholds established by different slice thickness and reconstruction algorithms in fast-kilovoltage-switching DECT scanners. It is important to evaluate the quantification consistency of IC values, because the current application of iodine maps is mainly based on established iodine concentration thresholds [2–4]. Our study suggested that the DLIR can be safely accepted as a new reconstruction algorithm for quantitative analysis of abdomen.

The thicker slice images have low image noise, but usually presents with lower spatial resolution and suffer from partial volume effects. This led to difficulties in displaying small and low-density objects. In contrast, the improved ERS and spatial resolution can provide higher sharpness and better contrast to allow better detectability of lesions. However, the thinner slice images have potential for improving the conspicuity for these lesions, but increase the image noise [9]. In our study, the thin slice AV-50 showed higher ERS values compared to thick slice AV-50, but suffered from the increased image noise, which resulted in suboptimal clinical acceptance evaluated by five radiologists. As the thinner slice thickness with AV-50 cannot provide satisfied balanced image quality for lesion detection, the new DLIR algorithm was introduced. To overcome the dilemma of spatial resolution and image noise, the DLIR algorithm was used and presented potential for improving image quality in VMIs [10–18]. The DLIR algorithm is developed by using deep convolutional neural networks with a ground-truth training data of filtered back-projection images acquired with high-dose scans, to generate high quality images from low-dose scans. The reduced image noise is believed to yield lower variability in the measured IC values [8]. The thin slice DLIR images presented similar ERS values compared to thin slice AV-50, while maintained relatively low image noise compared to thick slice AV-50, which gained higher clinical acceptance in subjective evaluation. Therefore, we believed that the DLIR may facilitate a thinner slice thickness as a new state-of-art standard for routine reconstruction of iodine maps in abdominal DECT, to replace the original thicker slice iodine maps using Asir-V.

The lesion conspicuity has been seldomly investigated in the iodine maps [8], while the studies using VMIs have demonstrated the potential of DLIR for improvement of lesion conspicuity [6, 7]. Our study suggested a possible improvement in lesion conspicuity in iodine maps by using DLIR, indicating a potential role of iodine maps for clinical diagnosis purpose in the future, in addition to the current iodine quantification. The thick slice AV-50 images, although with lower noise, were not optimal for diagnosis purpose due to thick slice thickness and lower sharpness. Cao et al. [9] have suggested in their study of using conventional CT images, the DLIR allows the use of thinner slice images by significantly suppressing image noise while improving image spatial resolution as well as overall image quality. Our study extended that statement into the iodine maps and recommended the thinner slice images as a new standard for iodine maps in abdominal DECT. The thin slice AV-50 images provided improved sharpness but suffered from high image noise, which potentially hindered the diagnosis. Xu et al. [13, 16] suggested that the DLIR significantly reduces image noise than Asir-V in low-keV VMIs, and were most evident and consistent in thin slice images. Sato et al. [7] and Noda et al. [11] showed representative cases for better lesion conspicuity in iodine maps using DLIR. Our study suggested small but significant improvements in lesion conspicuity using DLIR-M than AV-50, but the DLIR-H did not show significant advantages than AV-50. It is not surprising that the DLIR-M gained a higher rating in lesion conspicuity than DLIR-H in our study, since the DLIR-M images were preferred by the readers in subjective image quality evaluation and gained higher diagnostic acceptance. However, strength level selection of DLIR may depends on the clinical tasks, as previous studies recommended different strength level of DLIR for solid or cystic lesions [7, 18] and pancreatic cancers [11]. Our study has confirmed the ability of DLIR for improving image quality as well as lesion conspicuity in iodine maps by using objective and subjective evaluations. Nevertheless, comprehensive evaluation of abdominal diseases and modification of reconstruction parameters are needed before the iodine maps can be accepted as a new extra reference for diagnosis purpose.

Our study has following limitations to address. First, the current study was conducted with a relatively small sample size at one institution. Although *post hoc* power calculation showed high efficiency, our conclusions require further validation in other centers. Second, our study only employed only one fast-kilovoltage-switching DECT scanner since the DLIR algorithm is vendor-specific, and we only compared the vendor-specific Asir-V algorithm with DLIR-M and DLIR-H. The inter-vendor and inter-scanner differences were not assessed [5, 24–27]. However, we chose thick slice AV-50 iodine maps as the benchmark, to present the improvement accomplished by DLIR compared to the current clinical standard. The DLIR with low strength was not included because it is not hopeful to provide available image quality [17, 18]. Third, our study only measured the IC values of normal structures. The influence of DLIR on iodine quantification and diagnosis must be ascertained with respect to different diseases. Also, the potential influence of DLIR on advanced quantitative analysis was not evaluated [5, 25]. Fourth, the qualitative image evaluation in our study was conducted by five radiologists with 1- to 6-year-experience in radiology. The experience in radiologists may introduce bias in the rating. The results of our study should be validated by more studies with more radiologists with different levels of experience. Fifth, the diagnostic acceptance of using iodine maps was not compared with that of the VMIs, as the best kiloelectron voltage level for VMIs using DLIR has not been determined yet. Further comparisons between VMIs and iodine maps are necessary to tell whether iodine maps have potential advantages for diagnosis purpose. Sixth, the potential influence of factors like patient motion, contrast agent dosage, and scanner settings on image quality were not assessed in our study. The future study may focus on these factors to deepen the DLIR application in clinical practice. Finally, the DLIR algorithm is a black box. We need further investigation to gain acceptance in clinical practice. Further investigations on the its robustness to artifacts and noise [28–31], as well as its protentional influence on the later images processing steps [32–34]. The future investigations are encouraged to explore the impact of DLIR on specific types of lesions or comparing its performance across different patient populations. Moreover, the cost-effectiveness of implementing DLIR in clinical practice would also be an interesting topic.

To summarize, the thinner slice thickness iodine maps with DLIR in abdominal DECT can keep the iodine concentration measurement values unchanged with lower variability compared with the standard reconstructions to allow consistent quantitative iodine analysis using established threshold values, and can provide improved image quality with reduced image noise, more natural image texture, and better spatial resolution. Compared to the standard thicker slice reconstructions, the thinner slice thickness iodine images with DLIR have the potential can potentially improve the accuracy of lesion detection and characterization in abdominal DECT. Future studies are encouraged to determine whether DLIR has clinical impact on iodine quantification and diagnosis confidence for specific clinical tasks.

### Electronic supplementary material

Below is the link to the electronic supplementary material.

Supplementary Material 1

## Data Availability

The datasets used and/or analysed during the current study are available from the corresponding author on reasonable request.

## Abbreviations

Asir-V: Adaptive statistical iterative reconstruction-V
CV: Coefficient of variation
DECT: Dual-energy computed tomography
DLIR: Deep learning image reconstruction
ERS: Edge rise slope
IC: Iodine concentration
ICC: Intra-class correlation coefficient
NPS: Noise power spectrum
ROI: Region of interest
SD: Standard deviation
VMI: Virtual monochromatic image
