# Supplementary material for: Deep learning image reconstruction generates thinner slice iodine maps with improved image quality to increase diagnostic acceptance and lesion conspicuity: a prospective study on abdominal dual-energy CT
Source: BMC Med Imaging. 2024 Jun 26;24:159. doi: 10.1186/s12880-024-01334-0 (PMC11201298; doi:10.1186/s12880-024-01334-0)

**Supplementary Materials**

**Title:** Deep learning image reconstruction generates thinner slice iodine maps in abdominal dual-energy CT with improved image quality to increase diagnostic acceptance and lesion conspicuity: a prospective study with five readers

**List of Supplementary Materials**

Supplementary Note S1 Pilot study

Supplementary Note S2 Quantitative image analysis methods

Supplementary Note S3 Qualitative image analysis methods

Supplementary Note S4 Statistical analysis methods

Supplementary Table S1 Detail of included lesions for assessment

Supplementary Table S2 Intra- and inter-rater agreement of measurements and ratings

Supplementary Table S3 Effect size of quantitative evaluation metrics between different reconstruction algorithms

Supplementary Table S4 Effect size of qualitative evaluation metrics between different reconstruction algorithms

Supplementary Figure 1 Four examples of abdominal CT studies with measurements

**Supplementary Note S1 Pilot study**

Before the formal study, we performed a pilot study to generate and confirm our hypothesis, and test the feasibility of the measuring methodology. In our pilot study, we randomly chose 10 participants who were scheduled to undergo abdominal contrast-enhanced CT scan at our institution in the clinical practice. The raw data of portal-venous phase scan was reconstructed into four series of iodine maps for each participant: 5-mm and 1.25-mm with Asir-V at 50% blending (AV-50), and 1.25-mm with DLIR at low (DLIR-L), medium (DLIR-M), and high strength (DLIR-H), all with a standard kernel. Therefore, 10 participants × 5 reconstruction algorithms = 50 images series were generated in the pilot study.

A radiologist with 4 years of total experience in CT image interpretation measured the SD of liver IC and ERS values. The detailed measuring methodology is available in Supplementary Note S2 and S3. The mean ± standard deviation of the SD of liver IC, and ERS were as follows.

| Metrics | 5-mm AV-50 | 1.25-mm AV-50 | 1.25-mm DLIR-L | 1.25-mm DLIR-M | 1.25-mm DLIR-H |
| --- | --- | --- | --- | --- | --- |
| SD of liver IC, mgI/mL | 0.131 ± 0.020 | 0.255 ± 0.044^*^ | 0.256 ± 0.037 | 0.207 ± 0.032 | 0.155 ± 0.025 |
| ERS, (mgI/mL)/mm | 5.981 ± 1.606^*^ | 8.795 ± 2.722 | 8.973 ± 2.863 | 9.128 ± 2.917 | 8.705 ± 3.495 |

^*^The 1.25-mm AV-50 was measured as the reference level for SD of liver IC, and the 5-mm AV-50 was measured as the reference level for ERS.

According to the pilot results of SD of liver IC, the 1.25-mm AV-50 images have the highest noise, and the 5-mm AV-50 images have the lowest noise. Compared to 1.25-mm AV-50, 1.25-mm DLIR-M (paired t-test, P<0.001), and 1.25-mm DLIR-H (P<0.001) can provide lower noise. The However, the 1.25-mm DLIR-H can provide lower noise than 1.25-mm DLIR-M (P<0.001). 1.25-mm DLIR-L showed comparable level of noise to 1.25-mm AV-50 (P=0.725).

According to the pilot results of ERS, the 5-mm AV-50 images have the lowest spatial resolution, and the 1.25-mm images have the higher spatial resolution. Compared to 5-mm AV-50, all 1.25-mm DLIR-L (P=0.003), 1.25-mm DLIR-M (P=0.002), and 1.25-mm DLIR-H (P=0.017) can provide higher spatial resolution compared to 5-mm AV-50. The 1.25-mm DLIR image did not show better spatial resolution that that of 1.25-mm AV-50 images (all P>0.05). The significant difference between 1.25-mm DLIR images were not found (all P>0.05).

The 1.25-mm DLIR-L images were not reconstructed in our formal study because it can only provide better spatial resolution, but not expected to provide lower image noise. Therefore, our formal study hypothesized that the 1.25-mm DLIR-M and 1.25-mm DLIR-H images has potential in improving image quality (both image noise and spatial resolution) and further assessed their diagnostic confidence.

**Supplementary Note S2 Quantitative image analysis methods**

The quantitative image analysis was performed using imQuest software version 7.1 (Duke University; <https://deckard.duhs.duke.edu/~samei/tg233.html>) by a radiologist with 4 years of total experience in CT image interpretation. This radiologist has experience in using this software for measurements. Regions of interest (ROI) were selected on the 5 mm AV-50 images, and corresponding images were linked to make sure identical ROIs were used for the same anatomic structure of different reconstructions. The measurement of ten randomly selected participants were repeated by the same radiologist two weeks after the first readout, and by another radiologist with 5 years of total experience in CT image interpretation. These repeated measurements were used to calculate the intra- in inter-rater quantitative measurement variabilities, respectively.

**Iodine concentration (IC) and variability measurement**

The iodine concentration (IC) and its standard deviation (SD) of nine anatomic structures were measured: liver, spleen, pancreas, kidney, abdominal aorta (AO), main portal vein (MPV), inferior vena cava (IVC), subcutaneous fat in the anterior abdominal wall, and psoas muscle. Each IC and SD value was calculated by averaging the three-time measurements (six times in total for two kidneys, and psoas muscle of two sides). The coefficient of variation (CV) was also calculated to evaluate the extent of variability by dividing the mean IC by the SD (<https://doi.org/10.2214/AJR.22.27753>; <https://doi.org/10.1007/s00330-022-09127-1>; <https://doi.org/10.1007/s00330-021-08121-3>). Here are examples for IC and variability measurement of nine anatomic sites.

The ROIs remained unchanged among images series using four reconstruction algorithms in the same participant. ROI placements in the vasculature were performed while avoiding the vessel wall, calcifications, thrombi, and artifacts. ROI placements in the abdominal parenchymal organs were performed while avoiding the blood vessels, bile ducts, pancreatic duct, focal lesions, and artifacts. The measuring of liver, spleen, pancreas, and kidney were performed at the slice with the largest presentation of the organ, respectively. The measuring of AO was performed at the slice of celiac trunk level. The measuring of IVC at the slice of renal vein level. The measuring of MPV was performed at the slice of porta hepatis level. The measuring of subcutaneous fat in the anterior abdominal wall and psoas muscle were performed at the level of L1-2 vertebra.

The CV of liver in 5-mm AV-50 iodine maps, as an example, was calculated as: CV_liver_ = SD_liver_ / IC_liver_ = 1.44 / 18.76 = 0.077.

**Noise power spectrum (NPS) measurement**

The image noise magnitude was evaluated using noise power spectrum (NPS) by placing ROIs on the relatively homogenous region of the liver (<https://doi.org/10.1002/mp.15558>). To quantify the changes of magnitude and texture of the image noise, the NPS curve, noise, noise peak, and the average and the peak spatial frequency were automatically generated. The ROIs kept at the same location among four iodine maps with different reconstruction algorithms for each participant. Here is an example for NPS measurement.

**Edge rise slope (ERS) measurement**

The image sharpness was evaluated using edge rise slope (ERS), which was defined as the ratio of the IC difference between the last dip and the first peak on the rapid rising IC curve divided by the distance between the two points (<https://doi.org/10.1007/s00330-022-09146-y>). The ERS was measured using a selected axial plane that presents a running portal vein. Larger ERS numbers indicate sharper edges. Here is an example for ERS measurement. The ERS was calculated as: ERS = (Y_peak_- Y_dip_) / (X_peak_- X_dip_) = (46 - 20) / (15.09 – 11.85) = 8.02

**Supplementary Note S3 Qualitative image analysis methods**

The qualitative image quality was assessed by five experienced radiologists with 1 to 6 years of total experience in CT image interpretation. The readers independently assessed all images using a 5-point grading scale for image contrast, image noise, image sharpness, image texture, small structure visibility, diagnostic acceptability, and lesion conspicuity. The readers were instructed that a value of less than 3 was deemed unsatisfactory for clinical use. The readers were told what lesions to rate in each participant, but should detect it by themselves. All the images were presented to the readers randomly blinded to the reconstruction parameters, with a default window width of 15.0 mgI/mL and window level of 5.0 mgI/mL, using the same settings for daily image interpretation at the reading room. Since the slice thickness of the images were visually detectable, the slice thickness was not blinded to the readers. However, the image reconstruction algorithms were blinded to the readers. The readers were allowed to adjust the window width and level and viewing distance at will, and had no time limits for their evaluation. A radiologist with 5 years of total experience in CT image interpretation repeated all the images and lesions two weeks after the first readout. The intra- in inter-rater qualitative assessment variabilities were calculated using assessments of five raters and the repeated assessments by this rater, respectively.

All rating was performed using monitors (MDCC-4430, BRACO Co. Ltd., Belgium) with the same settings (system firmware version, v1.02.00; native resolution/pixel format 2560 × 1600; physical size, 654 × 409 mm; luminance 600 cd/m^2^) employed for daily image interpretation at the reading room (ambient light, less than 10 lx). The images were presented to the readers in a random fashion blinded to the reconstruction parameters, with a default window width of 15.0 mgI/mL and window level of 5.0 mgI/mL. The readers were allowed to adjust the window width and level and viewing distance as they preferred and had no time limits to complete the image review.

The definitions of image quality and lesion conspicuity rating are as follows (<https://doi.org/10.1007/s00330-021-08121-3>; <https://doi.org/10.1007/s00330-022-08647-0>; <https://doi.org/10.1007/s00330-022-09146-y>; <https://doi.org/10.1016/j.crad.2021.10.014>; <https://doi.org/10.1186/s13244-022-01308-2>; <https://doi.org/10.1007/s00330-022-09018-5>; <https://doi.org/10.1259/bjr.20211163>; <https://doi.org/10.1097/RCT.0000000000001316>; <https://doi.org/10.1007/s00261-023-03845-w>; <https://doi.org/10.1007/s10278-023-00806-z>; <https://doi.org/10.1007/s00330-023-09556-6>).

| **Rating** | **Image contrast** | **Image noise** | **Image sharpness** | **Image texture** | **Small structure visibility** | **Diagnostic acceptability** | **Lesion conspicuity** |
| --- | --- | --- | --- | --- | --- | --- | --- |
| 1 | very poor, undesired contrast affecting diagnostic confidence | Unacceptable, severe noise/ nondiagnostic | substantial blurring of structures/nondiagnostic | Over-smoothing, undesired texture affecting diagnostic confidence | Unacceptable, nondiagnostic conspicuity | Non-diagnostic; poor delineation of anatomic structures and non-diagnostic image quality | Probably absent, poor visualization of lesion from surrounding tissue |
| 3 | Acceptable contrast not affecting diagnostic confidence | Moderate, acceptable noise | Blurring of liver and vessel edges but not affecting diagnostic confidence | Average, acceptable texture/not affecting diagnostic confidence | Acceptable conspicuity | Satisfactory, good delineation of anatomic structures and diagnostic image quality | Equivocal, distinct lesion not clearly identified |
| 5 | Excellent contrast facilitating image assessment | Minimal, excellent image with low noise | Excellent quality with sharp contours of solid organ and in-plane vascular structures like hepatic veins edges | Realistic, optimal/excellent texture facilitating image assessment | Excellent conspicuity of adrenal glands and small vessels like hepatic, intra-renal, and gastroduodenal arteries | Excellent; sharp delineation of anatomical structures and excellent diagnostic image quality | Definitely present, unequivocal visualization of lesion distinct from surrounding tissue |

Here is an example of iodine maps reconstructed using portal-venous phase data of abdominal contrast-enhanced DECT. These iodine maps were reconstructed using the four different image reconstruction algorithms: 5-mm AV-50, 1.25-mm AV-50, 1.25-mm DLIR-L, 1.25-mm DLIR-M, and 1.25-mm DLIR-H, respectively. Note that the reconstruction parameters blinded to the readers during the readout.

**Supplementary Note S4 Statistical analysis methods**

**Comparisons with post hoc and subgroup analysis**

The repeated-measure analysis of variance and Friedman test were performed to compare the quantitative and qualitative metrics among the reconstruction algorithms, respectively. The *post hoc* pairwise comparisons were conducted with Bonferroni correction if a significant difference was found. For the lesion conspicuity, subgroup analysis was performed according to location of lesion (kidney versus liver versus others), largest diameter of lesion (<median versus >=median; median = 11 mm), and relative CT attenuation to surrounding tissue (lower versus higher or mixed). The statistical analysis with a two-tailed p<0.05 was considered as with statistical significance. The p values for post hoc analysis were presented as adjusted p values, i. e., the p values were multiplied by six, and the cutoff for statistical significance remained to be 0.05.

**Intra- and inter-rater agreement**

The quantitative image measurement of ten randomly selected participants were repeated by the same radiologist with 4 years of total experience in CT image interpretation two weeks after the first readout, and by another radiologist with 5 years of total experience in CT image interpretation. These repeated measurements were used to calculate the intra- in inter-rater quantitative measurement variabilities, respectively. The intra- and inter-rater agreements for quantitative analysis were assessed using intraclass correlation coefficient (ICC) with single measurement or rater, absolute agreement, two-way random effects model. The ICC values were interpreted as poor (<0.50), moderate (0.50–0.75), good (0.75-0.90), or excellent (≥0.90) (<https://doi.org/10.1016/j.jcm.2016.02.012>).

The qualitative rating were conducted by five radiologists with 1 to 6 years of total experience in CT image interpretation. A radiologist with 5 years of total experience in CT image interpretation repeated all the assessment of images and lesions two weeks after the first readout. The intra- in inter-rater qualitative assessment variabilities were calculated using assessments of five raters and the repeated assessments by this rater, respectively. The intra- and inter-rater agreements for qualitative analysis were evaluated using weighted kappa statistics and Kendall's W statistics. The weighted kappa statistic and Kendall's W statistic were interpreted as poor (<0.20), fair (0.20-0.40), moderate (0.40-0.60), good (0.60–0.80), and excellent (≥0.80) (<https://doi.org/10.1016/j.sapharm.2012.04.004>).

***A priori* sample size estimation**

The formula for sample size calculation (<https://doi.org/10.11613/BM.2021.010502>):

$$N=\frac{{(r+1)(Z_{\alpha/2}+Z_{1-\beta})}^{2}\sigma^{2}}{rd^{2}}$$

r - the ratio of sample size

σ – pooled standard deviation

d – difference of means of two groups

Z_α/2_ – 1.96 for alpha 0.05

Z_1-β_ – 1.04 for power 0.85

The 1.25-mm and 5-mm AV-50, and 1.25-mm DLIR-M and DLIR-H iodine maps were selected for *a priori* power calculation. The SD values of liver IC of 1.25-mm AV-50 and 1.25-mm DLIR-M and DLIR-H were selected for *a priori* sample size estimation, because 1.25-mm DLIR-M and DLIR-H was expected to provide reduced image noise of iodine maps when the thin slice thickness was used. The ERS values of 5-mm AV-50 and 1.25-mm DLIR-M and DLIR-H were selected for *a priori* sample size estimation, because 1.25-mm DLIR-M and DLIR-H was expected to provide higher spatial resolution of iodine maps when similar image noise was achieved by the 5-mm AV-50, the clinical reference standard at our institution. A beta value > 0.85 was considered as efficient power, when alpha was set at 0.05. The results in the pilot study were applied for calculation.

Results for *a priori* sample size estimation:

| Comparing groups | r | σ | d | Z_α/2_ | N | Z_1-β_ | 1-β | β |
| --- | --- | --- | --- | --- | --- | --- | --- | --- |
| 1.25-mm AV-50 vs 1.25-mm DLIR-H  SD of liver IC, mgI/mL | 1 | 0.062 | 0.100 | 1.96 | 7 | 1.04 | 0.15 | 0.85 |
| 5-mm AV-50 vs 1.25-mm DLIR-H  ERS, (mgI/mL)/mm | 1 | 2.994 | 2.724 | 1.96 | 22 | 1.04 | 0.15 | 0.85 |
| 1.25-mm AV-50 vs 1.25-mm DLIR-M  SD of liver IC, mgI/mL | 1 | 0.047 | 0.048 | 1.96 | 17 | 1.04 | 0.15 | 0.85 |
| 5-mm AV-50 vs 1.25-mm DLIR-M  ERS, (mgI/mL)/mm | 1 | 2.803 | 3.147 | 1.96 | 14 | 1.04 | 0.15 | 0.85 |

According to our preliminary study, the *a priori* sample size estimation yielded a size of 22 patients for a power of 0.85, when alpha was 0.05. Our study actually included 104 participants, which was expected to provide an efficient statistical power.

***Post hoc* power calculation**

The formula for sample size calculation (<https://doi.org/10.11613/BM.2021.010502>):

$$N=\frac{{(r+1)(Z_{\alpha/2}+Z_{1-\beta})}^{2}\sigma^{2}}{rd^{2}}$$

Therefore, the formula for sample power calculation is as follows:

$$Z_{1-\beta}=\sqrt{\frac{Nr}{r+1}}\times\frac{d}{\sigma}-Z_{\alpha/2}$$

Then, the β value was obtained cording to the normal distribution.

r - the ratio of sample size

σ – pooled standard deviation

d – difference of means of two groups

Z_α/2_ – 1.96 for alpha 0.05

Z_1-β_ – 0.84 for power 0.80, 1.04 for power 0.85, 1.28 for power 0.90, 1.64 for power 0.95

The 1.25-mm and 5-mm AV-50 and 1.25-mm DLIR-M and DLIR-H iodine maps were selected for *post hoc* power calculation. The SD values of liver IC of 1.25-mm AV-50 and 1.25-mm DLIR-M and DLIR-H were selected for *post hoc* power calculation, because 1.25-mm DLIR-M and DLIR-H was expected to provide reduced image noise of iodine maps when the thin slice thickness was used. The ERS values of 5-mm AV-50 and 1.25-mm DLIR-M and DLIR-H were selected for *post hoc* power calculation, because 1.25-mm DLIR-M and DLIR-H was expected to provide higher spatial resolution of iodine maps when similar image noise was achieved by the 5-mm AV-50, the clinical reference standard at our institution. A beta value > 0.85 was considered as efficient power, when alpha was set at 0.05. The results in the formal study were applied for calculation.

Results for *post hoc* power calculation:

| Comparing groups | r | σ | d | Z_α/2_ | N | Z_1-β_ | 1-β | β |
| --- | --- | --- | --- | --- | --- | --- | --- | --- |
| 1.25-mm AV-50 vs 1.25-mm DLIR-H  SD of liver IC, mgI/mL | 1 | 0.045 | 0.052 | 1.96 | 104 | 6.373 | >0.9999 | <0.0001 |
| 5-mm AV-50 vs 1.25-mm DLIR-H  ERS, (mgI/mL)/mm | 1 | 3.810 | 2.400 | 1.96 | 104 | 2.582 | 0.9952 | 0.0048 |
| 1.25-mm AV-50 vs 1.25-mm DLIR-M  SD of liver IC, mgI/mL | 1 | 0.046 | 0.043 | 1.96 | 104 | 4.781 | >0.9999 | <0.0001 |
| 5-mm AV-50 vs 1.25-mm DLIR-M  ERS, (mgI/mL)/mm | 1 | 3.805 | 2.429 | 1.96 | 104 | 2.643 | 0.9959 | 0.0041 |

The *post hoc* power calculation using sample size and key metrics (SD values of liver IC, and ERS), resulted in 1-beta values >0.99, when alpha was 0.05, indicating an efficient statistical power.

**Supplementary Table S1 Detail of included lesions for assessment**

| No. | Location | Largest diameter | Relative CT attenuation to surrounding tissue |
| --- | --- | --- | --- |
| 1 | Gallbladder | 19 | Mixed |
| 2 | Gallbladder | 4 | Higher |
| 3 | Gallbladder | 6 | Higher |
| 4 | Gallbladder | 7 | Higher |
| 5 | Left kidney | 3 | Lower |
| 6 | Left kidney | 4 | Lower |
| 7 | Left kidney | 5 | Lower |
| 8 | Left kidney | 5 | Lower |
| 9 | Left kidney | 5 | Lower |
| 10 | Left kidney | 5 | Lower |
| 11 | Left kidney | 5 | Lower |
| 12 | Left kidney | 5 | Lower |
| 13 | Left kidney | 6 | Lower |
| 14 | Left kidney | 7 | Lower |
| 15 | Left kidney | 7 | Lower |
| 16 | Left kidney | 7 | Lower |
| 17 | Left kidney | 8 | Lower |
| 18 | Left kidney | 8 | Lower |
| 19 | Left kidney | 10 | Lower |
| 20 | Left kidney | 11 | Lower |
| 21 | Left kidney | 12 | Mixed |
| 22 | Left kidney | 13 | Lower |
| 23 | Left kidney | 15 | Lower |
| 24 | Left kidney | 22 | Lower |
| 25 | Left kidney | 24 | Lower |
| 26 | Left kidney | 24 | Lower |
| 27 | Left kidney | 27 | Mixed |
| 28 | Left kidney | 31 | Lower |
| 29 | Left liver lobe | 4 | Lower |
| 30 | Left liver lobe | 5 | Lower |
| 31 | Left liver lobe | 5 | Lower |
| 32 | Left liver lobe | 8 | Lower |
| 33 | Left liver lobe | 9 | Lower |
| 34 | Left liver lobe | 11 | Lower |
| 35 | Left liver lobe | 11 | Lower |
| 36 | Left liver lobe | 12 | Mixed |
| 37 | Left liver lobe | 14 | Lower |
| 38 | Left liver lobe | 14 | Lower |
| 39 | Left liver lobe | 14 | Lower |
| 40 | Left liver lobe | 14 | Lower |
| 41 | Left liver lobe | 14 | Lower |
| 42 | Left liver lobe | 14 | Lower |
| 43 | Left liver lobe | 15 | Mixed |
| 44 | Left liver lobe | 23 | Mixed |
| 45 | Left liver lobe | 26 | Lower |
| 46 | Left liver lobe | 40 | Mixed |
| 47 | Left liver lobe | 107 | Lower |
| 48 | Pancreas | 19 | Lower |
| 49 | Pancreas | 23 | Lower |
| 50 | Right kidney | 4 | Lower |
| 51 | Right kidney | 4 | Lower |
| 52 | Right kidney | 4 | Lower |
| 53 | Right kidney | 5 | Lower |
| 54 | Right kidney | 5 | Lower |
| 55 | Right kidney | 5 | Lower |
| 56 | Right kidney | 5 | Lower |
| 57 | Right kidney | 5 | Lower |
| 58 | Right kidney | 6 | Lower |
| 59 | Right kidney | 6 | Lower |
| 60 | Right kidney | 6 | Lower |
| 61 | Right kidney | 6 | Lower |
| 62 | Right kidney | 7 | Lower |
| 63 | Right kidney | 7 | Lower |
| 64 | Right kidney | 8 | Lower |
| 65 | Right kidney | 8 | Lower |
| 66 | Right kidney | 8 | Lower |
| 67 | Right kidney | 8 | Lower |
| 68 | Right kidney | 8 | Lower |
| 69 | Right kidney | 9 | Lower |
| 70 | Right kidney | 10 | Lower |
| 71 | Right kidney | 10 | Lower |
| 72 | Right kidney | 11 | Lower |
| 73 | Right kidney | 12 | Lower |
| 74 | Right kidney | 12 | Lower |
| 75 | Right kidney | 12 | Lower |
| 76 | Right kidney | 13 | Lower |
| 77 | Right kidney | 13 | Lower |
| 78 | Right kidney | 17 | Lower |
| 79 | Right kidney | 26 | Lower |
| 80 | Right kidney | 42 | Lower |
| 81 | Right kidney | 53 | Lower |
| 82 | Right liver lobe | 3 | Lower |
| 83 | Right liver lobe | 4 | Lower |
| 84 | Right liver lobe | 4 | Lower |
| 85 | Right liver lobe | 5 | Higher |
| 86 | Right liver lobe | 5 | Lower |
| 87 | Right liver lobe | 5 | Lower |
| 88 | Right liver lobe | 5 | Lower |
| 89 | Right liver lobe | 5 | Lower |
| 90 | Right liver lobe | 5 | Lower |
| 91 | Right liver lobe | 6 | Lower |
| 92 | Right liver lobe | 6 | Lower |
| 93 | Right liver lobe | 6 | Lower |
| 94 | Right liver lobe | 7 | Higher |
| 95 | Right liver lobe | 7 | Lower |
| 96 | Right liver lobe | 8 | Lower |
| 97 | Right liver lobe | 9 | Higher |
| 98 | Right liver lobe | 9 | Lower |
| 99 | Right liver lobe | 9 | Lower |
| 100 | Right liver lobe | 10 | Lower |
| 101 | Right liver lobe | 11 | Lower |
| 102 | Right liver lobe | 11 | Lower |
| 103 | Right liver lobe | 12 | Lower |
| 104 | Right liver lobe | 12 | Lower |
| 105 | Right liver lobe | 12 | Lower |
| 106 | Right liver lobe | 12 | Lower |
| 107 | Right liver lobe | 13 | Higher |
| 108 | Right liver lobe | 13 | Lower |
| 109 | Right liver lobe | 13 | Lower |
| 110 | Right liver lobe | 13 | Mixed |
| 111 | Right liver lobe | 14 | Lower |
| 112 | Right liver lobe | 14 | Mixed |
| 113 | Right liver lobe | 14 | Mixed |
| 114 | Right liver lobe | 15 | Lower |
| 115 | Right liver lobe | 17 | Higher |
| 116 | Right liver lobe | 17 | Lower |
| 117 | Right liver lobe | 17 | Mixed |
| 118 | Right liver lobe | 18 | Lower |
| 119 | Right liver lobe | 18 | Lower |
| 120 | Right liver lobe | 21 | Mixed |
| 121 | Right liver lobe | 22 | Mixed |
| 122 | Right liver lobe | 23 | Higher |
| 123 | Right liver lobe | 23 | Lower |
| 124 | Right liver lobe | 30 | Mixed |
| 125 | Right liver lobe | 33 | Lower |
| 126 | Right liver lobe | 35 | Lower |
| 127 | Right liver lobe | 37 | Lower |
| 128 | Right liver lobe | 49 | Lower |
| 129 | Right liver lobe | 69 | Mixed |
| 130 | Spleen | 6 | Lower |
| 131 | Spleen | 9 | Lower |
| 132 | Spleen | 15 | Lower |
| 133 | Spleen | 23 | Lower |
| 134 | Stomach | 32 | Mixed |
| 135 | Stomach | 39 | Mixed |
| 136 | Stomach | 66 | Mixed |

**Supplementary Table S2 Intra- and inter-rater agreement of measurements and ratings**

| Metrics | Intra-rater agreement | Inter-rater agreement |
| --- | --- | --- |
| Quantitative measurements | ICC | ICC |
| IC of liver | 0.939 | 0.938 |
| SD of liver IC | 0.967 | 0.921 |
| Noise | 0.960 | 0.758 |
| Noise peak | 0.830 | 0.737 |
| f_peak_ | 0.891 | 0.809 |
| f_average_ | 0.901 | 0.731 |
| ERS | 0.933 | 0.964 |
| Qualitative measurements | Weighted kappa | Kendall's W |
| Image contrast | 0.624 | 0.651 |
| Image noise | 0.769 | 0.717 |
| Image sharpness | 0.656 | 0.536 |
| Image texture | 0.683 | 0.665 |
| Small structure visibility | 0.672 | 0.636 |
| Diagnostic acceptability | 0.641 | 0.574 |
| Lesion conspicuity | 0.546 | 0.551 |

**Supplementary Table S3 Effect size of quantitative evaluation metrics between different reconstruction algorithms**

|  | 5-mm AV-50  versus  1.25-mm AV-50 | 5-mm AV-50  versus  1.25-mm DLIR-M | 5-mm AV-50  versus  1.25-mm DLIR-H | 1.25-mm AV-50  versus  1.25-mm DLIR-M | 1.25-mm AV-50  versus  1.25-mm DLIR-H | 1.25-mm DLIR-M  versus  1.25-mm DLIR-H |
| --- | --- | --- | --- | --- | --- | --- |
| Iodine concentration and variability |  |  |  |  |  |  |
| Liver |  |  |  |  |  |  |
| IC, mgI/mL | 0.111 ± 0.424 | 0.101 ± 0.394 | 0.109 ± 0.351 | -0.01 ± 0.123 | -0.002 ± 0.186 | 0.008 ± 0.158 |
| SD, mgI/mL | -1.074 ± 0.332 | -0.647 ± 0.261 | -0.15 ± 0.246 | 0.427 ± 0.138 | 0.924 ± 0.226 | 0.498 ± 0.133 |
| CV | -0.044 ± 0.014 | -0.027 ± 0.011 | -0.006 ± 0.010 | 0.018 ± 0.006 | 0.038 ± 0.011 | 0.02 ± 0.006 |
| Spleen |  |  |  |  |  |  |
| IC, mgI/mL | 0.226 ± 1.971 | 0.312 ± 2.987 | 0.061 ± 0.392 | 0.086 ± 3.557 | -0.165 ± 1.992 | -0.25 ± 2.958 |
| SD, mgI/mL | -1.062 ± 0.321 | -0.6 ± 0.301 | -0.122 ± 0.289 | 0.462 ± 0.152 | 0.940 ± 0.240 | 0.478 ± 0.132 |
| CV | -0.035 ± 0.022 | -0.022 ± 0.038 | -0.004 ± 0.009 | 0.013 ± 0.042 | 0.031 ± 0.022 | 0.018 ± 0.037 |
| Pancreas |  |  |  |  |  |  |
| IC, mgI/mL | -0.123 ± 1.138 | -0.100 ± 1.115 | -0.112 ± 1.081 | 0.022 ± 0.219 | 0.011 ± 0.279 | -0.011 ± 0.241 |
| SD, mgI/mL | -1.206 ± 0.663 | -0.921 ± 0.570 | -0.358 ± 0.483 | 0.285 ± 0.300 | 0.849 ± 0.453 | 0.564 ± 0.308 |
| CV | -0.049 ± 0.031 | -0.037 ± 0.026 | -0.014 ± 0.020 | 0.012 ± 0.014 | 0.034 ± 0.021 | 0.022 ± 0.014 |
| Kidney |  |  |  |  |  |  |
| IC, mgI/mL | -0.224 ± 1.753 | -0.252 ± 1.646 | -0.181 ± 1.945 | -0.028 ± 0.320 | 0.043 ± 1.126 | 0.071 ± 1.065 |
| SD, mgI/mL | -0.779 ± 0.852 | -0.521 ± 0.823 | -0.412 ± 0.245 | 0.258 ± 0.285 | -0.633 ± 0.245 | -0.891 ± 0.245 |
| CV | -0.012 ± 0.014 | -0.008 ± 0.014 | -0.047 ± 0.478 | 0.004 ± 0.005 | -0.034 ± 0.479 | -0.038 ± 0.479 |
| Abdominal aorta |  |  |  |  |  |  |
| IC, mgI/mL | 0.600 ± 1.470 | 0.664 ± 0.999 | 0.669 ± 0.975 | 0.064 ± 1.032 | 0.069 ± 1.006 | 0.005 ± 0.250 |
| SD, mgI/mL | -0.305 ± 0.479 | -0.637 ± 0.272 | -0.343 ± 0.407 | -0.332 ± 0.274 | 0.961 ± 0.275 | 0.293 ± 0.234 |
| CV | -0.391 ± 3.716 | -0.092 ± 0.738 | -0.007 ± 0.007 | 0.299 ± 0.795 | 0.383 ± 0.716 | 0.084 ± 0.737 |
| Main portal vein |  |  |  |  |  |  |
| IC, mgI/mL | 0.764 ± 1.366 | 0.604 ± 1.337 | 0.672 ± 1.525 | -0.16 ± 0.244 | -0.092 ± 1.067 | 0.068 ± 1.023 |
| SD, mgI/mL | -1.326 ± 0.666 | -1.025 ± 0.608 | -0.451 ± 0.598 | 0.301 ± 0.236 | 0.875 ± 0.394 | 0.574 ± 0.334 |
| CV | -0.025 ± 0.013 | -0.019 ± 0.011 | -0.008 ± 0.011 | 0.006 ± 0.005 | 0.017 ± 0.008 | 0.011 ± 0.007 |
| Inferior vena cava |  |  |  |  |  |  |
| IC, mgI/mL | 0.616 ± 1.055 | 0.561 ± 1.012 | 0.769 ± 2.136 | -0.055 ± 0.270 | 0.153 ± 1.916 | 0.209 ± 1.956 |
| SD, mgI/mL | -1.209 ± 0.551 | -0.881 ± 0.511 | -0.312 ± 0.443 | 0.327 ± 0.321 | 0.897 ± 0.393 | 0.570 ± 0.275 |
| CV | -0.032 ± 0.018 | -0.023 ± 0.016 | -0.01 ± 0.025 | 0.009 ± 0.008 | 0.022 ± 0.019 | 0.013 ± 0.017 |
| Psoas major |  |  |  |  |  |  |
| IC, mgI/mL | -0.216 ± 0.450 | -0.173 ± 0.431 | -0.140 ± 0.418 | 0.043 ± 0.119 | 0.077 ± 0.225 | 0.034 ± 0.181 |
| SD, mgI/mL | -0.985 ± 0.242 | -0.528 ± 0.189 | -0.048 ± 0.165 | 0.457 ± 0.159 | 0.937 ± 0.211 | 0.480 ± 0.091 |
| CV | -0.149 ± 0.063 | -0.077 ± 0.044 | -0.002 ± 0.036 | 0.072 ± 0.036 | 0.148 ± 0.054 | 0.076 ± 0.023 |
| Abdominal subcutaneous fat |  |  |  |  |  |  |
| IC, mgI/mL | 8.341 ± 1.175 | 0.613 ± 1.315 | 0.359 ± 2.484 | -7.728 ± 2.102 | -7.982 ± 2.210 | -0.253 ± 2.353 |
| SD, mgI/mL | -2.182 ± 0.646 | -0.538 ± 0.436 | -0.162 ± 0.391 | 1.644 ± 0.368 | 2.020 ± 0.365 | 0.376 ± 0.214 |
| CV | 0.178 ± 1.125 | 0.042 ± 0.104 | 0.004 ± 0.105 | -0.136 ± 1.125 | -0.175 ± 1.125 | -0.039 ± 0.050 |
| Noise, mgI/mL | -1.158 ± 0.199 | -0.718 ± 0.179 | -0.222 ± 0.175 | 0.439 ± 0.095 | 0.936 ± 0.139 | 0.496 ± 0.080 |
| Noise peak， (mgI/mL)^2^ · mm^2^ | -15.83 ± 7.086 | -5.202 ± 3.922 | -0.711 ± 3.187 | 10.627 ± 4.062 | 15.119 ± 5.523 | 4.491 ± 1.730 |
| f_peak_, mm^-1^ | -0.011 ± 0.030 | -0.020 ± 0.036 | -0.014 ± 0.036 | -0.009 ± 0.025 | -0.002 ± 0.028 | 0.007 ± 0.013 |
| f_average_, mm^-1^ | -0.012 ± 0.025 | -0.027 ± 0.023 | -0.022 ± 0.023 | -0.016 ± 0.015 | -0.011 ± 0.017 | 0.005 ± 0.011 |
| ERS, (mgI/mL)/mm | -2.294 ± 3.362 | -2.428 ± 3.362 | -2.400 ± 3.369 | -0.134 ± 1.038 | -0.106 ± 1.182 | 0.029 ± 0.567 |

Note: Data were presented as mean ± standard deviation.

**Supplementary Table S4 Effect size of qualitative evaluation metrics between different reconstruction algorithms**

|  | 5-mm AV-50  versus  1.25-mm AV-50 | 5-mm AV-50  versus  1.25-mm DLIR-M | 5-mm AV-50  versus  1.25-mm DLIR-H | 1.25-mm AV-50  versus  1.25-mm DLIR-M | 1.25-mm AV-50  versus  1.25-mm DLIR-H | 1.25-mm DLIR-M  versus  1.25-mm DLIR-H |
| --- | --- | --- | --- | --- | --- | --- |
| Image contrast | 1.354 ± 0.209 | -0.335 ± 0.302 | 0.413 ± 0.272 | -1.688 ± 0.269 | -0.940 ± 0.212 | 0.748 ± 0.225 |
| Image noise | -1.044 ± 0.167 | -1.265 ± 0.312 | -1.521 ± 0.192 | -0.221 ± 0.294 | -0.477 ± 0.172 | -0.256 ± 0.313 |
| Image sharpness | -1.092 ± 0.316 | -0.794 ± 0.358 | -1.440 ± 0.338 | 0.298 ± 0.330 | -0.348 ± 0.228 | -0.646 ± 0.368 |
| Image texture | -0.923 ± 0.170 | -1.296 ± 0.255 | -1.306 ± 0.251 | -0.373 ± 0.270 | -0.383 ± 0.232 | -0.010 ± 0.290 |
| Small structure visibility | -0.767 ± 0.279 | -1.006 ± 0.286 | -1.321 ± 0.266 | -0.238 ± 0.319 | -0.554 ± 0.273 | -0.315 ± 0.273 |
| Diagnostic acceptability | 1.354 ± 0.209 | -0.335 ± 0.302 | 0.413 ± 0.272 | -1.688 ± 0.269 | -0.940 ± 0.212 | 0.748 ± 0.225 |
| Lesion conspicuity | -0.899 ± 0.349 | -1.044 ± 0.577 | -0.932 ± 0.367 | -0.146 ± 0.444 | -0.034 ± 0.199 | 0.112 ± 0.463 |

Note: Data were presented as mean ± standard deviation. N=136 for lesion conspicuity.

**Supplementary Figure 1 Four examples of abdominal CT studies with measurements**

These portal-venous phase abdominal CT studies were reconstructed into iodine maps using 5-mm AV-50, 1.25-mm AV-50, 1.25-mm DLIR-M, and 1.25-mm DLIR-H algorithms, respectively; and present with the same windowing (width of 15.0 mgI/mL, level of 5.0 mgI/mL).

(A) A patient was scanned for a suspected left renal lesion. With 1.25-mm DLIR-H images, the internal enhancement was better detected. The difference of iodine concentration between the low-density compartment and subtle enhancement was more obvious with 1.25-mm AV-50 (1.14 mgI/mL) and 1.25-mm DLIR-H (1.04 mgI/mL) images, than 5-mm AV-50 (0.68 mgI/mL) image, suggesting the malignancy of the lesion.

The measurements in 5-mm AV-50, 1.25-mm AV-50, and 1.25-mm DLIR-H.

5-mm AV-50: Delta = 11.00-4.22 = 6.78 = 0.678 mgI/mL

1.25-mm AV-50: Delta = 15.18-3.78 = 11.4 = 1.14 mgI/mL

1.25-mm DLIR-H: Delta = 14.00-3.56 = 10.44 = 1.04 mgI/mL


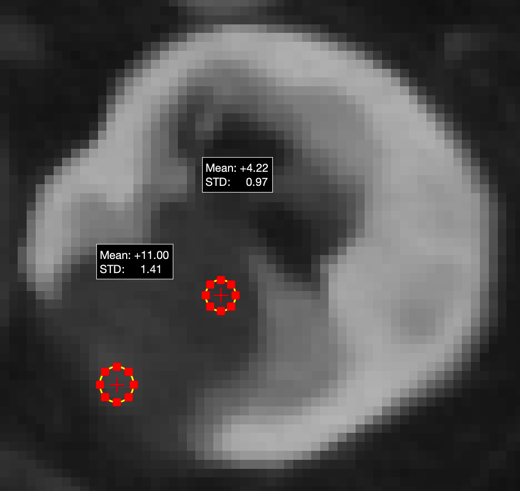

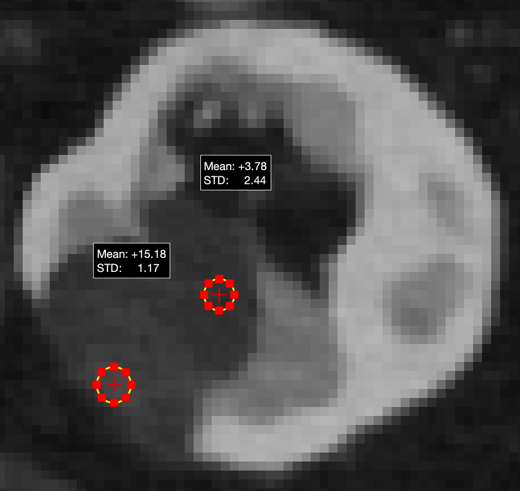

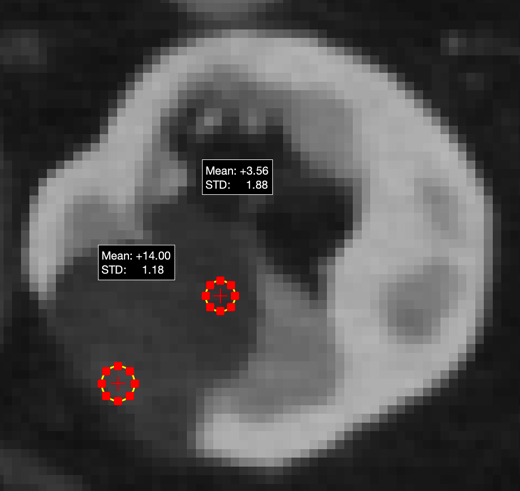


(B) A patient was scanned for cancer staging. The hepatic lesion with subtle circular and center enhancement in with 5-mm AV-50 image was suspected to be a metastasis lesion. The 1.25-mm images better visualized the boundary of lesion, and the subtle circular enhancement was excluded. The follow-up scans indicated that it is a benign lesion. The detailed center enhancement in the lesion can be better depicted in 1.25-mm images. The ERS of border of the enhancement was higher in 1.25-mm AV-50 [0.437 (mgI/mL)/mm] image than 5-mm AV-50 [0.262 (mgI/mL)/mm] image, and was even higher in 1.25 DLIR-H [0.655 (mgI/mL)/mm] image.

The measurements in 5-mm AV-50, 1.25-mm AV-50, and 1.25-mm DLIR-H.

5-mm AV-50: ERS = (22-16) / (14.84-12.55) = 2.62 = 0.262 (mgI/mL)/mm

1.25-mm AV-50: ERS = (24-14) / (14.84-12.55) = 4.37 = 0.437 (mgI/mL)/mm

1.25-mm DLIR-H: ERS = (27-12) / (14.84-12.55) = 6.55 = 0.655 (mgI/mL)/mm


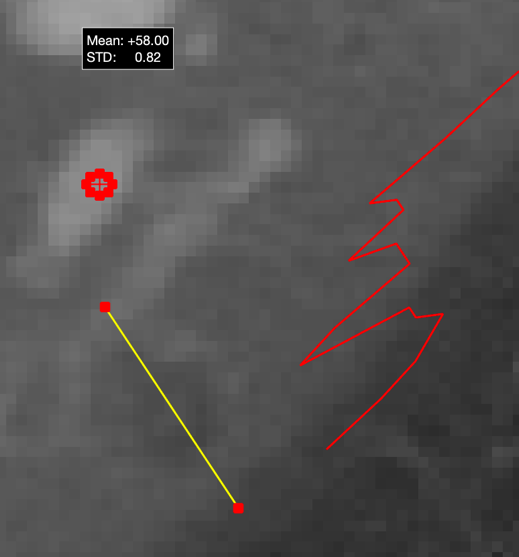

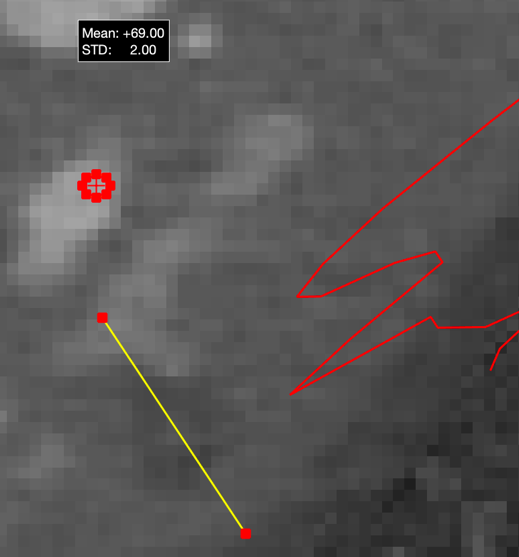

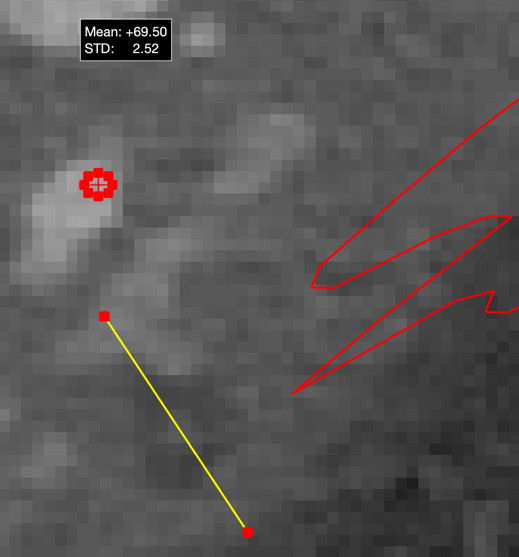


(C) A patient was scanned for a lesion in spleen. The difference of iodine concentration between the low-density lesion and spleen parenchyma was more obvious with 1.25-mm AV-50 (1.14 mgI/mL) and 1.25-mm DLIR-H (1.04 mgI/mL) images, than 5-mm AV-50 (0.60 mgI/mL) image. The ERS of the lesion boundary was higher in 1.25-mm AV-50 [0.334 (mgI/mL)/mm] and 1.25 DLIR-H [0.359 (mgI/mL)/mm] images than 5-mm AV-50 [0.164 (mgI/mL)/mm] image. The 1.25-mm DLIR-H image with higher contrast and clearer boundary improved the diagnostic confidence of a cyst.

The measurements in 5-mm AV-50, 1.25-mm AV-50, and 1.25-mm DLIR-H.

5-mm AV-50: Delta = 27.00-21.00 = 6.00 = 0.60 mgI/mL

1.25-mm AV-50: Delta = 29.75-18.33 = 11.42 = 1.14 mgI/mL

1.25-mm DLIR-H: Delta = 28.75-18.33 = 10.42 = 1.04 mgI/mL

5-mm AV-50: ERS = (28-20) / (14.59-9.724) = 1.64 = 0.164 (mgI/mL)/mm

1.25-mm AV-50: ERS = (27-14) / (14.59-10.7) = 3.34 = 0.334 (mgI/mL)/mm

1.25-mm DLIR-H: ERS = (28-14) / (14.59-10.7) = 3.59 = 0.359 (mgI/mL)/mm


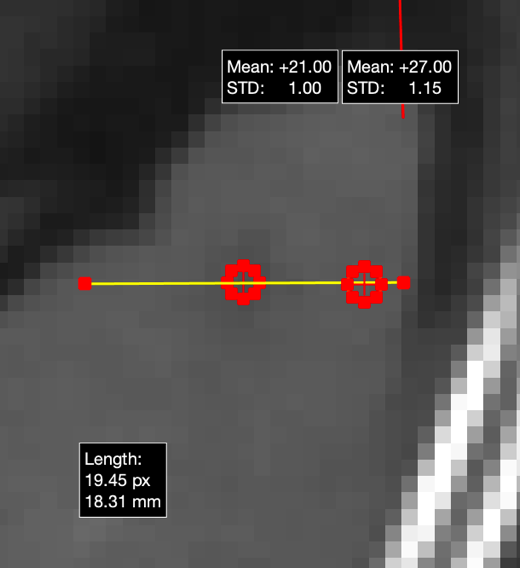

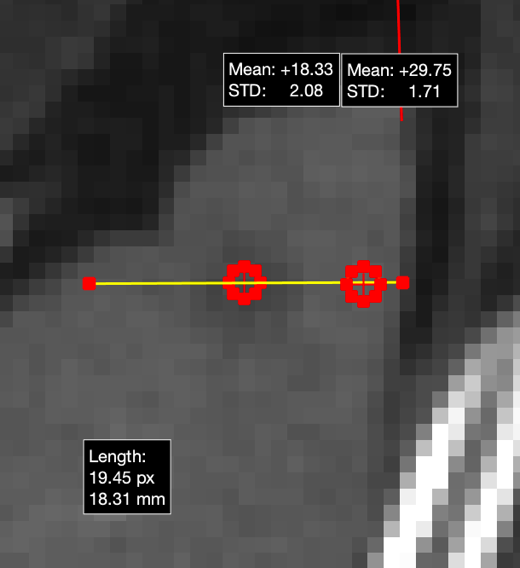

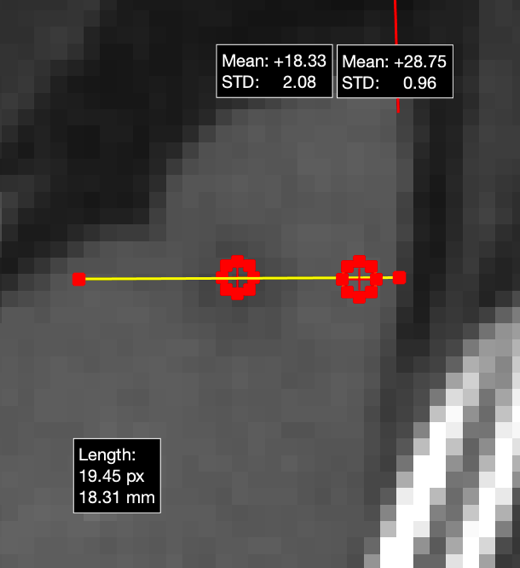


(D) A patient with gastric cancer was scanned for hepatic metastases. With the 1.25-mm images, the left boundary of the lesion was clearer than the 5-mm AV-50 images. The ERS of the subtle left boundary was higher in in 1.25-mm DLIR-H [0.586 (mgI/mL)/mm] image than 1.25 AV-50 [0.373 (mgI/mL)/mm] image, and lowest in 5-mm AV-50 [0.166 (mgI/mL)/mm] image. It was hard to measure the size of the lesion in 5-mm image, while it can be easier to be measured in 1.25-mm images, to guide later treatment selections.

The measurements in 5-mm AV-50, 1.25-mm AV-50, and 1.25-mm DLIR-H.

5-mm AV-50: ERS = (27-24) / (4.682-2.875) = 1.66 = 0.166 (mgI/mL)/mm

1.25-mm AV-50: ERS = (28-21) / (5.63-3.753) = 3.73 = 0.373 (mgI/mL)/mm

1.25-mm DLIR-H: ERS = (29-18) / (5.63-3.753) = 5.86 = 0.586 (mgI/mL)/mm


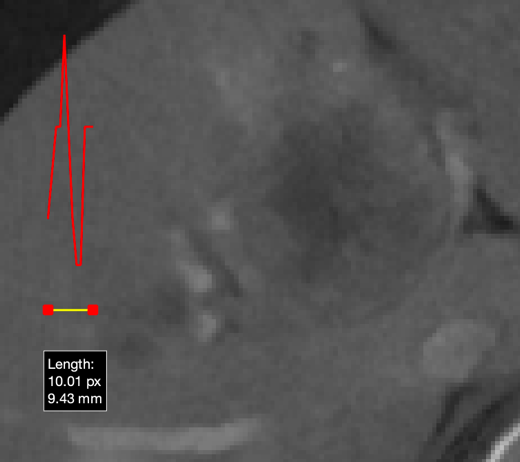

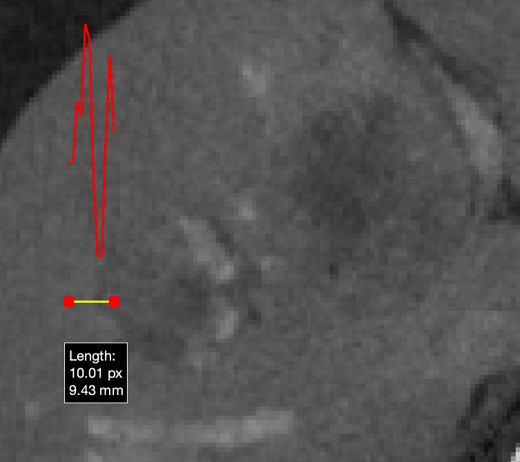

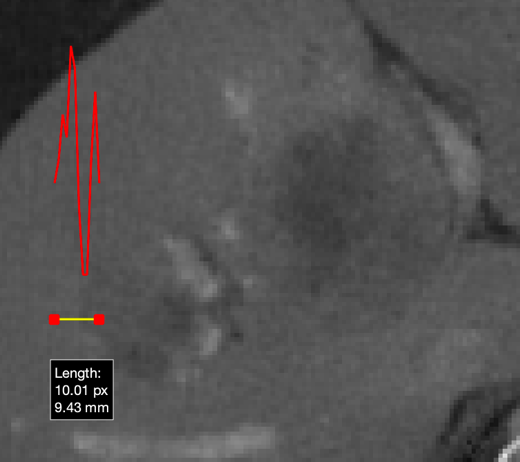

Supplement: Supplementary file 1 — Supplementary Material 1 [file 12880_2024_1334_MOESM1_ESM.docx]
